# Skipjack tuna bone-derived biocalcium ameliorates osteoblast and osteoclast differentiation through microRNA21 regulation

**DOI:** 10.1016/j.isci.2026.116808

**Published:** 2026-07-16

**Authors:** Sompot Jantarawong, Sudarat Phuntong, Saowapak Kanobthammakul, Papitchaya Watcharanurak, Jidapa Szekely, Jedsada Kaewrakmuk, Eakolarn Chotianuson, Doungporn Amornlerdpison, Chutima S. Vaddhanaphuti, Theeraphol Senphan, Yutthana Pengjam

**Affiliations:** 1 Faculty of Medical Technology, Prince of Songkla University, Songkhla 90110, Thailand; 2 Master of Business Administration Program in Innovation in Human Capital and Entrepreneurship Management, Suan Sunandha Rajabhat University, Dusit, Bangkok 10300, Thailand; 3 Center of Excellence in Agricultural Innovation for Graduate Entrepreneur, Maejo University, Sansai, Chiang Mai 50290, Thailand; 4 Faculty of Fisheries Technology and Aquatic Resources, Maejo University, Sansai, Chiang Mai 50290, Thailand; 5 Innovative Research Unit of Epithelial Transport and Regulation (iETR), Department of Physiology, Faculty of Medicine, Chiang Mai University, Chiang Mai 50200, Thailand; 6 Program in Food Science and Technology, Faculty of Engineering and Agro-industry, Maejo University, Sansai, Chiang Mai 50290, Thailand

**Keywords:** Skipjack tuna bone, bio-calcium, osteoblast, osteoclast, microRNA-21

## Abstract

Alternative calcium sources are needed to enhance osteoporosis management. This study investigated the osteoprotective effects of skipjack tuna bone-derived biocalcium (Bio). Bio enhanced MC3T3-E1 osteoblast and C3H10T1/2 cell differentiation, increasing alkaline phosphatase activity and calcium deposition. Conversely, Bio inhibited receptor activator of nuclear factor-κB ligand (RANKL)-induced osteoclastogenesis in RAW 264.7 macrophages, reducing tartrate-resistant acid phosphatase (TRAP) activity and bone resorption. Bio suppressed intracellular ROS and downregulated primary, precursor and mature microRNA (miR)-21. miR-21 inhibitor suppressed TRAP activity and Cathepsin K expression, as opposed to miR-21 mimic. In an osteoblast-osteoclast crosstalk environment, Bio synergistically enhanced miR-21 target genes (PTEN and PDCD4) expression, whereas PTEN knockdown suppressed NFATc1 osteoclast marker expression via miR-21 inhibition. Molecular docking confirmed binding affinities between mouse and human miR-21 and both targets. Western blot showed β-catenin upregulation and total NF-κB p65 suppression after Bio treatment. Collectively, Bio exerts dual anti-osteoporotic actions, offering a potential functional supplement for bone remodeling disorders.

## Highlights

• Skipjack tuna bone-derived biocalcium promotes osteoblast differentiation
• Biocalcium suppresses RANKL-induced osteoclastogenesis and bone resorption
• Biocalcium downregulates intracellular ROS and microRNA-21 in osteoclasts
• Osteoblast crosstalk enhances PTEN and PDCD4 expression in osteoclasts

## Graphical abstract

**Figure:**
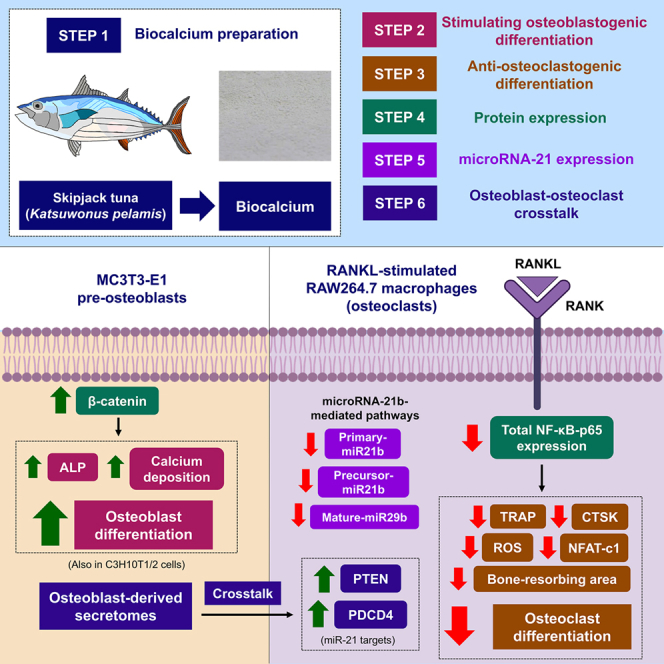

## Teaser

Molecular biology; Biotechnology

## Introduction

Bone is a dynamic tissue characterized by a continuous remodeling process maintained by the coupled activities of bone-forming osteoblasts and bone-resorbing osteoclasts.^1^^,^^2^ Osteoblasts, derived from mesenchymal precursors, secrete alkaline phosphatase (ALP) during bone matrix formation, and then promote mineralization through deposition of calcium phosphate (hydroxyapatite).^1^^,^^2^ In contrast, osteoclasts are multinucleated cells of hematopoietic progenitors that resorb bone by secreting acid and proteolytic enzymes (e.g., Cathepsin K [CTSK]).^1^^,^^2^ *In vitro*, receptor activator of nuclear factor-κB ligand (RANKL)-stimulated RAW264.7 macrophages serve as a standard osteoclastogenesis model: RANKL binding to RANK triggers canonical NF-κB signaling pathway, leading to the activation of transcription factor nuclear factor of activated T cells (NFATc1) and the upregulation of osteoclast-specific markers (e.g., tartrate-resistant acid phosphatase [TRAP] and CTSK).^3^^,^^4^ Maintaining the balance between bone formation and bone resorption is vital, as excessive resorption leads to the progressive bone loss characteristic of osteoporosis.^1^^,^^2^ Calcium is fundamental to this process, serving as a critical regulator of cell differentiation.^5^^,^^6^ However, typical dietary intakes often fall short of required levels,^7^^,^^8^ and many conventional supplements suffer from poor bioavailability,^9^ driving the need for more effective nutritional interventions.

Marine-derived resources, particularly by-products from the tuna processing industry, offer a sustainable and high-value alternative to traditional forms of calcium. Skipjack tuna (*Katsuwonus pelamis*) bones constitute approximately 10%–15% of the total biomass in the canning industry, and their repurposing into biocalcium (Bio) significantly reduces the environmental burden associated with seafood waste.^10^^,^^11^^,^^12^ Through sequential enzymatic and chemical treatments, these bones are converted into a high-purity hydroxyapatite complex. This material is characterized by a superior whiteness index (*L∗* value of 88.67) and an optimal mineral profile, containing approximately 23.02% calcium and 10.14% phosphorus.^10^^,^^11^^,^^12^ A defining functional advantage of skipjack tuna bone-derived Bio is its nature as an organic-mineral complex. Unlike inorganic salts, Bio retains a proteinaceous matrix rich in collagen, evidenced by high glycine (315 residues/1,000) and imino acid (179 residues/1,000) content.^10^^,^^11^^,^^12^ These associated peptides play a critical role in enhancing biological efficiency by chelating calcium ions, which prevents the precipitation and crystallization of insoluble calcium phosphate in the gastrointestinal tract.^10^^,^^11^^,^^12^ Consequently, Bio exhibits a superior bioavailability of approximately 13%, compared with the 10% typically observed in synthetic calcium carbonate.^10^^,^^11^^,^^12^ The systemic benefits of this Bio have been well-documented in preclinical models. In ovariectomized rats, Bio supplementation has been shown to effectively mitigate bone loss, increasing bone mineral density and improving trabecular architecture.^12^ Furthermore, recent molecular investigations have revealed that Bio acts as a potent regulator of key signaling pathways, including the suppression of proinflammatory cytokines (IL-6, TNF-α, and IL-1β) and the modulation of the microRNA (miR)-29b axis to preserve muscle integrity.^13^

miR-21 has been identified as a pivotal epigenetic modulator in bone remodeling, characterized by its complex dual influence on both osteoblasts and osteoclasts.^14^^,^^15^ In the osteogenic lineage, miR-21 acts as a positive regulator of differentiation, mineralization, and fracture healing by downregulating inhibitory protein, specially phosphatase and tensin homolog (PTEN).^14^^,^^15^ This interaction activates the PI3K/AKT signaling pathway, which ultimately enhances RUNX2 expression and the secretion of key osteogenic marker, including osteocalcin.^14^^,^^15^ Conversely, miR-21 facilitates RANKL-induced osteoclastogenesis and bone resorption by suppressing CTSK expression, post-transcriptionally silencing programmed cell death 4 (PDCD4), and targeting PTEN to activate the PI3K/AKT signaling pathway.^14^^,^^15^ Given its role as a molecular switch in the coupling of bone formation and resorption, the dysregulation of miR-21 is frequently implicated in the pathogenesis of bone disease, particularly osteoporosis.^4^^,^^16^^,^^17^^,^^18^ Consequently, understanding the modulation of miR-21 is essential for evaluating the therapeutic potential of novel interventions, such as Bio, in maintaining bone homeostasis. Despite these promising findings, the direct influence of such Bio interventions on the differentiation and crosstalk of osteoblasts and osteoclasts remains to be fully characterized *in vitro*.

Therefore, this study aimed to investigate the biomolecular and osteoprotective effects of skipjack tuna bone-derived Bio using mouse MC3T3-E1 osteoblasts and RANKL-stimulated RAW 264.7 macrophages. By evaluating ALP activity, mineral deposition, and TRAP activity alongside CTSK expression, we sought to determine if this marine-derived Bio can simultaneously stimulate osteogenesis and attenuate osteoclastogenesis. Crucially, the research explored the regulatory role of Bio on the intracellular ROS/miR-21 axis—encompassing primary, precursor, and mature forms of miR-21—and the subsequent modulation of target genes PTEN and PDCD4 within an osteoblast-osteoclast crosstalk environment. Molecular docking simulations and western blot analysis of β-catenin and NF-κB p65 signaling were further utilized to elucidate the underlying molecular mechanisms. This research provides comprehensive cellular and molecular evidence validating the potential of Bio as a sustainable and effective nutritional strategy for the management of osteoporosis.

## Results

### Bio upregulates osteoblast differentiation *in vitro*

**Figure 1:**
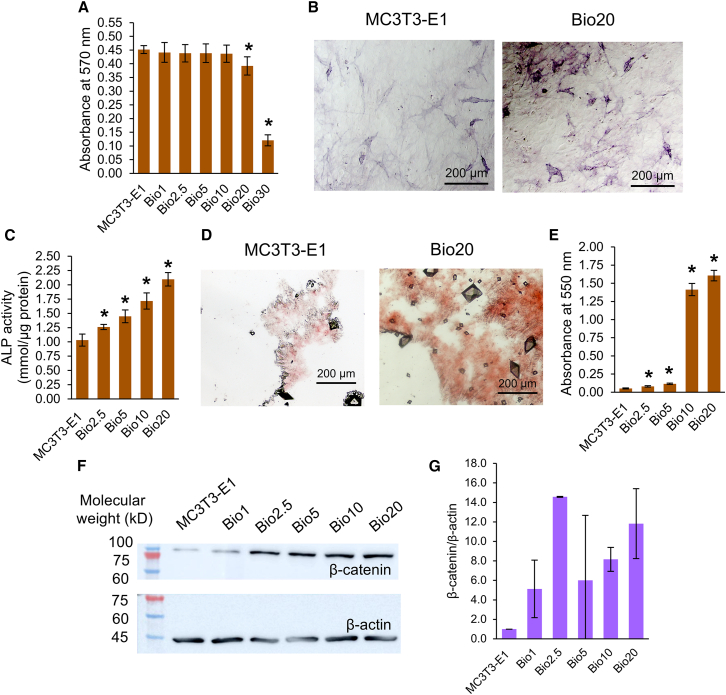
The regulation of biocalcium on the differentiation of MC3T3-E1 osteoblasts *in vitro* (A) Cell viability of MC3T3E-1 osteoblasts after treatment of skipjack tuna bone-derived bio-calcium (Bio). (B) Alkaline phosphatase (ALP) staining of MC3T3E-1 osteoblasts. (C) ALP activity of MC3T3E-1 osteoblasts after Bio treatment. (D) Alizarin red S staining of MC3T3E-1 osteoblasts. (E) Absorbance at 550 nm in Bio-treated MC3T3E-1 osteoblasts measured after Alizarin red S staining. (F) Cropped western blot images of β-catenin and β-actin in MC3T3-E1 osteoblasts. (G) Relative β-catenin expression in MC3T3-E1 osteoblasts. Cells were treated with Bio at concentrations of 1, 2.5, 5, 10, 20, and 30 μg/mL (Bio1, Bio2.5, Bio5, Bio10, Bio20, and Bio30, respectively). Data in bar charts are represented as mean ± SD. *n* = 8 for cell viability (MTT assay), *n* = 6 for ALP activity and absorbance at 550 nm measured after Alizarin red S staining, and *n* = 3 for others. *∗p* < 0.05 by Student’s *t* test (A, C, E, and G). (B and D) Scale bars represent 200 μm.

The effects of Bio on osteoblast viability and differentiation were evaluated using the MC3T3-E1 osteoblasts. Cell viability (MTT) assay demonstrated that treatment with Bio at 1–10 μg/mL showed no cytotoxic effects, whereas higher concentrations of 20 and 30 μg/mL significantly reduced cell viability (Figure 1A; Tables S1–S3; *p* values: Bio1 = 0.2658, Bio2.5 = 0.1653, Bio5 = 0.1787, Bio10 = 0.1619, Bio20 = 0.0013, and Bio30 = 2.9083 × 10^−9^).

To assess osteogenic differentiation, ALP activity and mineralization were evaluated. Bio significantly enhanced ALP activity in a dose-dependent manner (Figures 1B and 1C; Tables S4–S6; *p* values: Bio2.5 = 0.0012, Bio5 = 0.0015, Bio10 = 6.7266 × 10^−5^, and Bio20 = 7.8495 × 10^−6^). These results were corroborated by ALP staining, where the control did not show notable staining. In contrast, Bio at 20 μg/mL resulted in visibly increased numbers of ALP-positive (purple-stained) cells, indicating enhanced early-stage osteogenic differentiation.

Further, calcium mineral deposition was assessed via Alizarin Red S staining and quantification (Figures 1D and 1E; Tables S7–S9). MC3T3-E1 osteoblasts treated with Bio (2.5–20 μg/mL) exhibited significant, concentration-dependent increases in mineralization (*p* values: Bio2.5 = 0.0010, Bio5 = 7.7401 × 10^−5^, Bio10 = 1.0364 × 10^−7^, and Bio20 = 1.9073 × 10^−8^). At the highest tested dose (Bio at 20 μg/mL), Alizarin Red S staining revealed a visibly higher density of red-stained mineralized nodules compared with untreated control. Hence, these findings indicate that Bio effectively promote osteoblastic activity and mineralization *in vitro*.

The influence of skipjack tuna bone-derived Bio on the protein expression of β-catenin in MC3T3-E1 osteoblasts was evaluated using western blot analysis (Figures 1F, 1G, and S1–S6; Tables S10–S12). For β-catenin, which is a key marker for osteoblast differentiation and the Wnt signaling pathway, Bio treatment showed a tendency to increase its expression levels in a dose-dependent manner compared with the untreated control. Specifically, the relative expression increased to 5.13-, 14.57-, 6.01-, 8.16-, and 11.82-fold for Bio at 1, 2.5, 5, 10, and 20 μg/mL, respectively, although these increases did not reach statistical significance (*p* values: Bio1 = 0.3951, Bio2.5 = 0.2735, Bio5 = 0.3445, Bio10 = 0.3002, and Bio20 = 0.2738).

**Figure 2:**
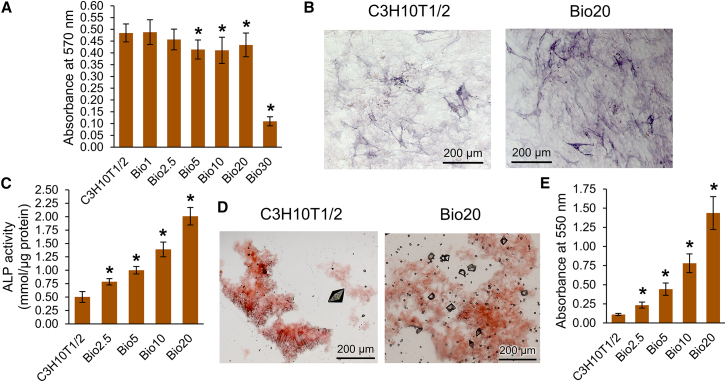
The regulation of biocalcium on the differentiation of C3H10T1/2 cells *in vitro* (A) Cell viability of C3H10T1/2 cells after treatment of skipjack tuna bone-derived bio-calcium (Bio). (B) Alkaline phosphatase (ALP) staining of C3H10T1/2 cells. (C) ALP activity of C3H10T1/2 cells after Bio treatment. (D) Alizarin red S staining of C3H10T1/2 cells. (E) Absorbance at 550 nm in Bio-treated C3H10T1/2 cells measured after Alizarin red S staining. Cells were treated with Bio at concentrations of 1, 2.5, 5, 10, 20, and 30 μg/mL (Bio1, Bio2.5, Bio5, Bio10, Bio20, and Bio30, respectively). Data in bar charts are represented as mean ± SD. *n* = 8 for cell viability (MTT assay), *n* = 6 for ALP activity and absorbance at 550 nm measured after Alizarin red S staining, and *n* = 3 for others. *∗p* < 0.05 by Mann-Whitney *U* test (A) and Student’s *t* test (C and E). (B and D) Scale bars represent 200 μm.

To further validate the osteogenic potential of Bio across different cellular models, parallel experiments were performed using C3H10T1/2 cells, a mouse multipotent mesenchymal stem cell line. The MTT assay revealed that Bio maintained a favorable cytocompatibility profile, exhibiting no observable cytotoxicity at lower concentrations while significantly decreasing cell survival only at 20 μg/mL of Bio (Figure 2A; Tables S13 and S14; *p* values: Bio1 = 0.7209, Bio2.5 = 0.1605, Bio5 = 0.0047, Bio10 = 0.0104, Bio20 = 0.0312, and Bio30 = 1.554 × 10^−4^), consistent with the patterns observed in MC3T3-E1 osteoblasts. Despite statistical significance indicated by *p* values at 5–20 μg/mL of Bio, Cohen’s *d* demonstrated that cell viability was vastly lower at 30 μg/mL, compared with lower Bio concentrations. Hence, 20 μg/mL of Bio is the maximum Bio concentration used in subsequent experiments. Furthermore, treatment with Bio triggered a significant, concentration-dependent upregulation in both enzymatic ALP activity and quantitative calcium mineral deposition (Figures 2B–2E; Tables S15–S20; *p* values of ALP activity: Bio2.5 = 2.2467 × 10^−4^, Bio5 = 5.0861 × 10^−5^, Bio10 = 1.3162 × 10^−5^, and Bio20 = 3.1753 × 10^−7^; *p* values of absorbance at 550 nm in Alizarin red S staining: Bio2.5 = 5.6982 × 10^−4^, Bio5 = 1.0478 × 10^−4^, Bio10 = 1.2775 × 10^−5^, and Bio20 = 9.8427 × 10^−6^). Taken together, these findings in C3H10T1/2 cells strongly corroborate the results of MC3T3-E1 osteoblasts, demonstrating that Bio consistently promotes mesenchymal precursor differentiation and extracellular matrix mineralization *in vitro*.

### Bio downregulates osteoclast differentiation *in vitro*

**Figure 3:**
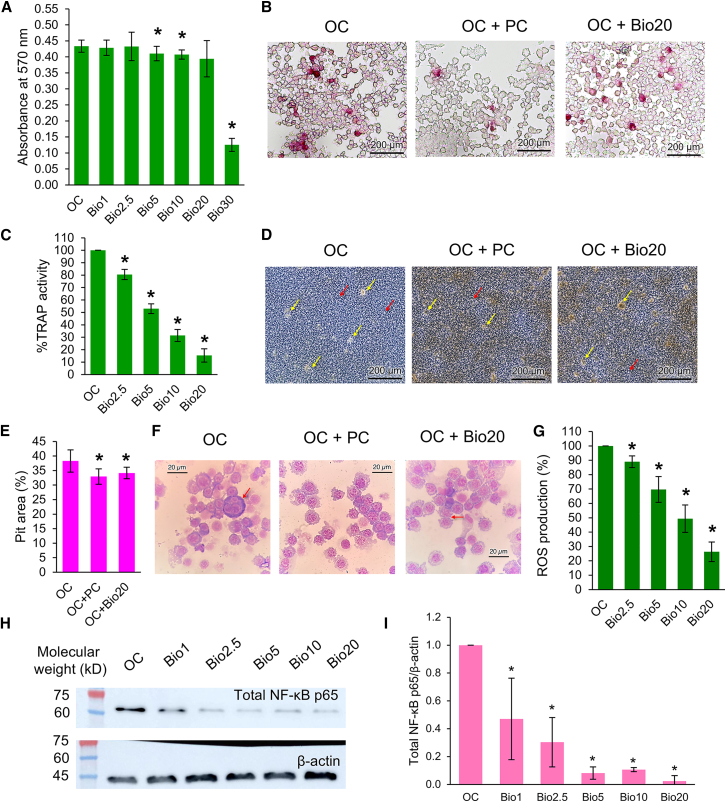
The regulation of biocalcium on the differentiation of osteoclasts *in vitro* (A) Cell viability after treatment of skipjack tuna bone-derived bio-calcium (Bio). (B) Tartrate-resistance acid phosphatase (TRAP) staining. (C) TRAP activity after Bio treatment. (D) Microscopy images examined using bone pit assay. (E) Percentage of pit area of microscopy images examined using bone pit assay. (F) Wright-Giemsa staining. (G) ROS production. (H) Cropped western blot images of total NF-κB p65 and β-actin in OC. (I) Relative total NF-κB p65 expression in osteoclasts (OC: RANKL-stimulated RAW264.7 macrophages). Cells were treated with Bio at concentrations of 1, 2.5, 5, 10, 20, and 30 μg/mL (Bio1, Bio2.5, Bio5, Bio10, Bio20, and Bio30, respectively). Symbols in (D): yellow arrows = OC; red arrows = pit area (indicating white resorption zones). Symbol in (F): red arrows = binucleated and multinucleated cells (cells containing at least three nuclei). PC = positive control (2 nM alendronate sodium trihydrate). Data in bar charts are represented as mean ± SD. *n* = 8 for cell viability (MTT assay) and *n* = 3 for others. *∗p* < 0.05 by Mann-Whitney *U* test (A) or Student’s *t* test (C, E, G, and I). (B and D) Scale bars represent 200 μm. (E) Scale bars represent 20 μm.

The effects of Bio on osteoclast viability and differentiation were examined using RANKL-stimulated RAW264.7 macrophages. Cell viability assay indicated that Bio at 1, 2.5, and 20 μg/mL had no cell viability effects, whereas concentrations of 5, 10, and 30 μg/mL resulted in significant viability reductions (Figure 3A; Tables S21 and S22; *p* values: Bio1 = 0.6629, Bio2.5 = 0.8089, Bio5 = 0.0415, Bio10 = 0.0023, Bio20 = 0.3227, and Bio30 = 7.77 × 10^−5^).

Osteoclast differentiation was further evaluated by TRAP staining and activity (Figures 3B and 3C; Tables S23–S25). TRAP staining showed that treatment with 2 nM alendronate sodium trihydrate (positive control) or Bio at 20 μg/mL reduced the number of TRAP-positive multinucleated cells (osteoclasts). Among these, treatment of positive control produced the most substantial suppression of TRAP staining. Consistent with the staining data, Bio at 2.5–20 μg/mL significantly suppressed TRAP activity in RANKL-stimulated RAW264.7 macrophages in a dose-dependent manner (*p* values: Bio2.5 = 0.0072, Bio5 = 0.0012, Bio10 = 8.1243 × 10^−4^, and Bio20 = 6.6463 × 10^−4^), indicating inhibition of osteoclast differentiation.

The functional consequence of this inhibition was further supported by the results of the bone resorption (pit) assay (Figures 3D and 3E; Tables S26–S28). Bio at 20 μg/mL markedly reduced the percentage of resorbed bone surface. Positive control, as expected, led to the greatest reduction in pit formation compared with Bio treatments (*p* values: OC + PC = 0.0154 and OC + Bio20 = 0.0378).

Wright-Giemsa staining (Figure 3F) confirmed the presence of binucleated and multinucleated osteoclasts (with ≥3 nuclei) in the control group, as well as in groups treated with Bio (20 μg/mL). In contrast, multinucleated osteoclasts were absent in the positive control-treated group, suggesting complete inhibition of osteoclast differentiation. These results demonstrate that while Bio does not completely inhibit osteoclastogenesis, it significantly attenuates osteoclast differentiation and bone resorptive activity in a concentration-dependent manner.

Bio treatment at concentrations ranging from 2.5 to 20 μg/mL significantly and dose-dependently reduced intracellular ROS levels compared to the RANKL-induced control group (Figure 3G; Tables S29–S31; *p* values: Bio2.5 = 5.7847 × 10^−4^, Bio5 = 2.0809 × 10^−4^, Bio10 = 2.4221 × 10^−5^, and Bio20 = 7.1141 × 10^−7^). Bio treatment significantly and dose-dependently downregulated the expression of total NF-κB p65, a critical mediator of inflammatory signaling in osteoclastogenesis. Compared with the control group, Bio at concentrations of 1, 2.5, 5, 10, and 20 μg/mL resulted in a significant reduction of total NF-κB p65 protein levels (Figures 3H, 3I, and S7–S12; Tables S32–S34; *p* values: Bio1 = 0.0443, Bio2.5 = 0.0104, Bio5 = 3.9011 × 10^−5^, Bio10 = 4.5905 × 10^−5^, and Bio10 = 2.6944 × 10^−4^). At 20 μg/mL of Bio, NF-κB expression was almost completely suppressed, indicating that Bio effectively inhibits the inflammatory pathway during the cellular response.

### Bio suppresses miR-21 expression

**Figure 4:**
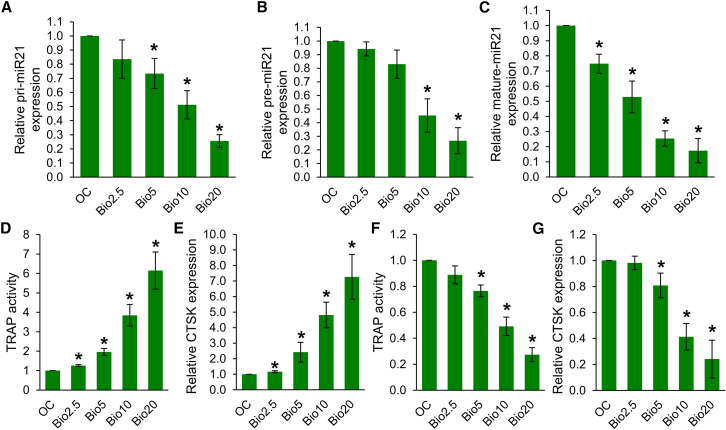
Modulation of the microRNA-21 signaling axis in osteoclasts by skipjack tuna bone-derived Bio (A) Relative expression of primary miR-21 (pri-miR-21). (B) Relative expression of precursor miR-21 (pre-miR-21). (C) Relative expression of mature-miR-21. (D and E) TRAP activity (D) and relative CTSK expression (E) in osteoclasts (OC: RANKL-stimulated RAW264.7 macrophages) following miR-21 mimic transfection. (F and G) TRAP activity (D) and relative CTSK expression (G) in OC following miR-21 inhibitor transfection. Cells were treated with Bio at concentrations of 2.5, 5, 10, and 20 μg/mL (Bio2.5, Bio5, Bio10, and Bio20, respectively). Data in bar charts are represented as mean ± SD. *n* = 3. *∗p* < 0.05 by Student’s *t* test.

To further explore the molecular regulatory role of miR-21 in osteoclastogenesis, quantitative RT-PCR was utilized to assess the expression of various forms of miR-21. Bio treatment resulted in a substantial and dose-dependent downregulation of primary- (pri-), precursor- (pre-), and mature-miR-21 (Figures 4A–4C; Tables S35–S43). For pri-miR-21, significant inhibitory effects were observed at higher concentrations (*p* values: Bio2.5 = 0.0857, Bio5 = 0.0248, Bio10 = 0.0068, and Bio20 = 5.8304 × 10^−4^). Pre-miR-21 expression was similarly suppressed (*p* values: Bio2.5 = 0.0941, Bio5 = 0.0528, Bio10 = 8.1214 × 10^−4^, and Bio20 = 0.0028), while the mature-miR-21 showed a significant dose-dependent decrease across all tested concentrations (*p* values: Bio2.5 = 0.0101, Bio5 = 0.0080, Bio10 = 7.7803 × 10^−4^, and Bio20 = 0.0015).

### Impact of miR-21 promotion and inhibition on Bio-mediated anti-osteoclastogenic effects

To confirm the functional involvement of miR-21 in the anti-osteoclastogenic activity of Bio, gain- and loss-of-function experiments were conducted by transfecting RANKL-stimulated RAW 264.7 macrophages with a specific miR-21 mimic and inhibitor, respectively (Figures 3D–3H; Tables S44–S55). In the presence of the miR-21 inhibitor, the addition of Bio continued to exhibit a potent, dose-dependent inhibitory effect on osteoclast function and gene expression. Specifically, Bio at concentrations of 5, 10, and 20 μg/mL significantly suppressed TRAP activity (*p* values: Bio2.5 = 0.0539, Bio5 = 0.0063, Bio10 = 0.0032, and Bio20 = 9.2673 × 10^−4^). Furthermore, the expression of the osteoclast-specific marker CTSK was markedly downregulated following Bio treatment in the transfected cells (*p* values: Bio2.5 = 0.2996, Bio5 = 0.0361, Bio10 = 0.0050, and Bio20 = 0.0061). Conversely, transient overexpression of miR-21 via the mimic significantly stimulated osteoclastogenesis, as evidenced by a baseline increase in both TRAP activity (*p* values: Bio2.5 = 0.0061, Bio5 = 0.0056, Bio10 = 0.0062, and Bio20 = 0.0056) and CTSK expression (*p* values: Bio2.5 = 0.0193, Bio5 = 0.0308, Bio10 = 0.0076, and Bio20 = 0.0085). These findings collectively demonstrate that Bio effectively attenuates osteoclast differentiation and proteolytic capacity, reinforcing its potential as a bioactive agent for regulating the miR-21 signaling axis during bone remodeling.

### Bio inhibits NFATc1 expression in PTEN-knockdown osteoclasts through miR-21 biogenesis

**Figure 5:**
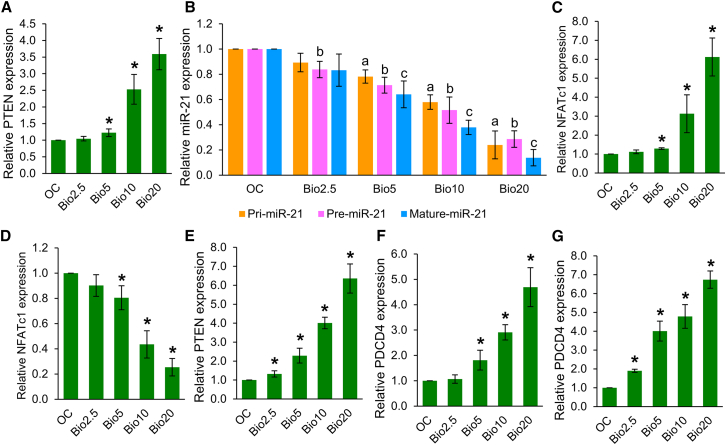
Regulation of miR-21 targets phosphatase and tensin homolog and programmed cell death 4 by biocalcium (A) Relative PTEN expression in osteoclasts (OC: RANKL-stimulated RAW264.7 macrophages) cultured under standard RANKL-supplemented differentiation medium. (B) Relative primary- (pri-), precursor- (pre-), and mature-miR-21 in PTEN-knockdown (KD) OC cultured under standard RANKL-supplemented differentiation medium. (C) Relative NFATc1 expression in PTEN-KD OC cultured under standard RANKL-supplemented differentiation medium and transfected with an miR-21 mimic. (D) Relative NFATc1 expression in PTEN-KD OC cultured under standard RANKL-supplemented differentiation medium and transfected with an miR-21 inhibitor. (E) Relative PTEN expression in OC cultured under standard medium enriched with 10% (*v*/*v*) osteoblast-conditioned medium (OCM). (F) Relative PDCD4 expression in OC cultured under standard RANKL-supplemented differentiation medium. (G) Relative PDCD4 expression in OC cultured under standard medium enriched with 10% (*v*/*v*) OCM. Cells were treated with skipjack tuna bone-derived biocalcium (Bio) at concentrations of 2.5, 5, 10, and 20 μg/mL (Bio2.5, Bio5, Bio10, and Bio20, respectively). Data in bar charts are represented as mean ± SD. *n* = 3. ^a, b, c^*p* < 0.05 compared with relative pri-, pre-, and mature-miR-21 expression in PTEN-KD OC cultured under standard RANKL-supplemented differentiation medium, respectively, by Student’s *t* test. *∗p* < 0.05 compared with Bio-untreated OC by Student’s *t* test.

To investigate whether the anti-osteoclastogenic mechanism of Bio is mediated through the miR-21 signaling axis, we examined the baseline impact of Bio treatment on the gene expression of the key downstream target, PTEN, in osteoclasts. qRT-PCR analysis demonstrated that Bio significantly upregulated relative PTEN expression in a dose-dependent manner compared with the Bio-untreated control group, with notable increases achieved at concentrations of 5, 10, and 20 μg/mL (Figure 5A; Tables S56–S58; *p* values: Bio2.5 = 0.1849, Bio5 = 0.0388, Bio10 = 0.0137, and Bio20 = 0.0054).

To further elucidate the downstream cascade, functional loss-of-function experiments were conducted using PTEN-knockdown (KD) osteoclasts. We assessed the transcriptional stages of miR-21 biogenesis, encompassing its pri-, pre-, and mature-miR-21. Bio treatment successfully drove a significant, concentration-dependent downregulation across all three molecular variants of miR-21 (pri-, pre-, and mature-miR-21) relative to the Bio-untreated PTEN-KD control group (Figure 5B; Tables S59–S67; *p* values of pri-miR-21: Bio2.5 = 0.0641, Bio5 = 0.0094, Bio10 = 0.0031, and Bio20 = 0.0035; *p* values of pre-miR-21: Bio2.5 = 0.0248, Bio5 = 0.0079, Bio10 = 0.0075, and Bio20 = 0.0014; *p* values of mature-miR-21: Bio2.5 = 0.0754, Bio5 = 0.0139, Bio10 = 0.0014, and Bio20 = 9.4066 × 10^−4^). To confirm the functional connection to downstream transcriptional signaling, the relative expression of the master osteoclastogenic regulator, NFATc1, was evaluated under opposite miR-21 transfection configurations. In PTEN-KD osteoclasts transfected with an miR-21 mimic—which naturally forces cell differentiation—Bio intervention systematically overrode this promotive effect, leading to a marked, dose-dependent upregulation of NFATc1 levels at higher doses (Figure 5C; Tables S68–S70; *p* values: Bio2.5 = 0.0915, Bio5 = 0.0040, Bio10 = 0.0329, and Bio20 = 0.0062). Conversely, when the cells were transfected with an miR-21 inhibitor to disrupt the signaling loop, Bio administration at concentrations of 5, 10, and 20 μg/mL induced a potent, concentration-dependent suppression of relative NFATc1 expression (Figure 5D; Tables S71–S73; *p* values: Bio2.5 = 0.0921, Bio5 = 0.0352, Bio10 = 0.0060, and Bio20 = 0.0014). Taken together, these findings collectively indicate that Bio effectively controls and limits osteoclast differentiation by suppressing downstream miR-21 biogenesis and modulating target factor pathways even within a PTEN-deficient microenvironment.

### Bio promotes PDCD4 and PTEN expression through osteoblast-osteoclast crosstalk

To investigate the intercellular regulatory effects of Bio within the bone microenvironment, the expression of PTEN and PDCD4—two critical downstream targets of miR-21—was analyzed in RANKL-stimulated RAW 264.7 macrophages under both standard RANKL-supplemented differentiation medium and standard medium enriched with 10% (*v*/*v*) osteoblast-conditioned medium (OCM) environments (Figures 5A and 5E–5G; Tables S56–S58 and S74–S82). Under standard RANKL-supplemented differentiation medium condition, Bio treatment significantly and dose-dependently upregulated PTEN expression (*p* values: Bio2.5 = 0.1849, Bio5 = 0.0388, Bio10 = 0.0137, and Bio20 = 0.0054) and PDCD4 (*p* values: Bio2.5 = 0.1246, Bio5 = 0.-025, Bio10 = 0.0161, and Bio20 = 0.0047). Remarkably, the supplementation of the culture environment with 10% *v*/*v* OCM substantially enhanced these effects, suggesting a synergistic interaction between Bio and osteoblast-derived secretomes. In this crosstalk environment, Bio induced a much more pronounced increase in PTEN levels (*p* values: Bio2.5 = 0.0386, Bio5 = 0.0146, Bio10 = 0.0017, and Bio20 = 0.0034) and PDCD4 expression (*p* values: Bio2.5 = 0.0014, Bio5 = 0.0051, Bio10 = 0.0046, and Bio20 = 0.0011) compared with the standard condition. These results indicate that skipjack tuna bone-derived Bio not only directly modulates osteoclastogenic inhibitors but also functions within a complex cellular network, where its efficacy is further amplified by factors secreted during osteoblastogenesis.

### Molecular docking of miR-21 with PTEN and PDCD4

**Figure 6:**
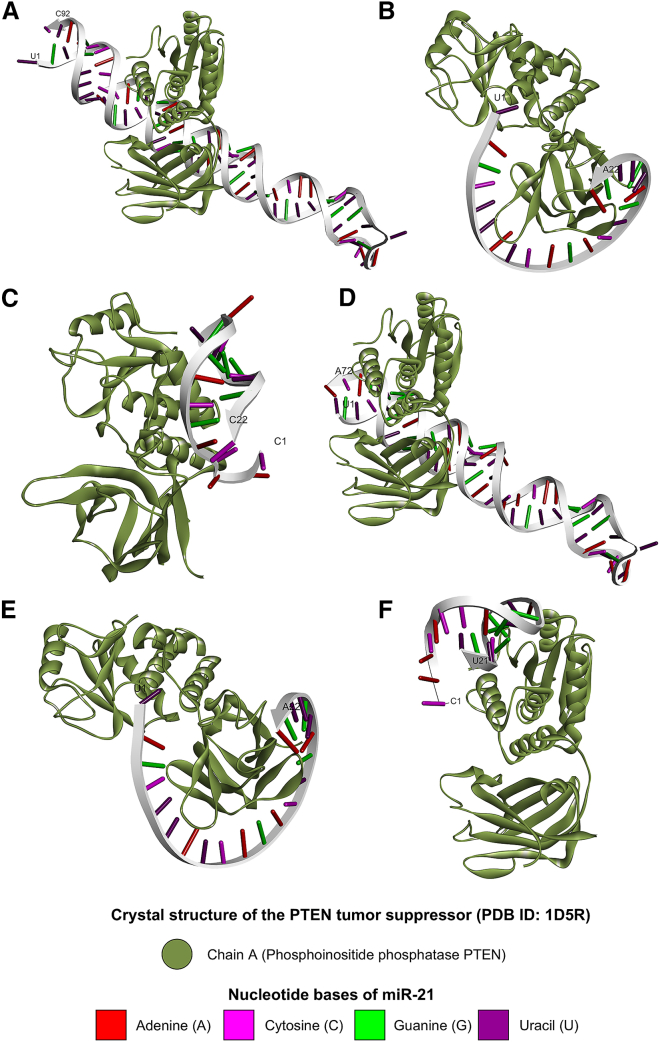
Predicted molecular docking interactions between miR-21 and PTEN (A) Stem-loop mmu-miR-21a. (B) Mature mmu-miR-21a-5p. (C) Mature mmu-miR-21a-3p. (D) Stem-loop hsa-miR-21. (E) Mature hsa-miR-21-5p. (F) Mature hsa-miR-21-3p. Inset labels denote the names and numbers of the first and last nucleotide bases of miR-21. All figures were generated using BIOVIA Discovery Studio Visualizer.

**Figure 7:**
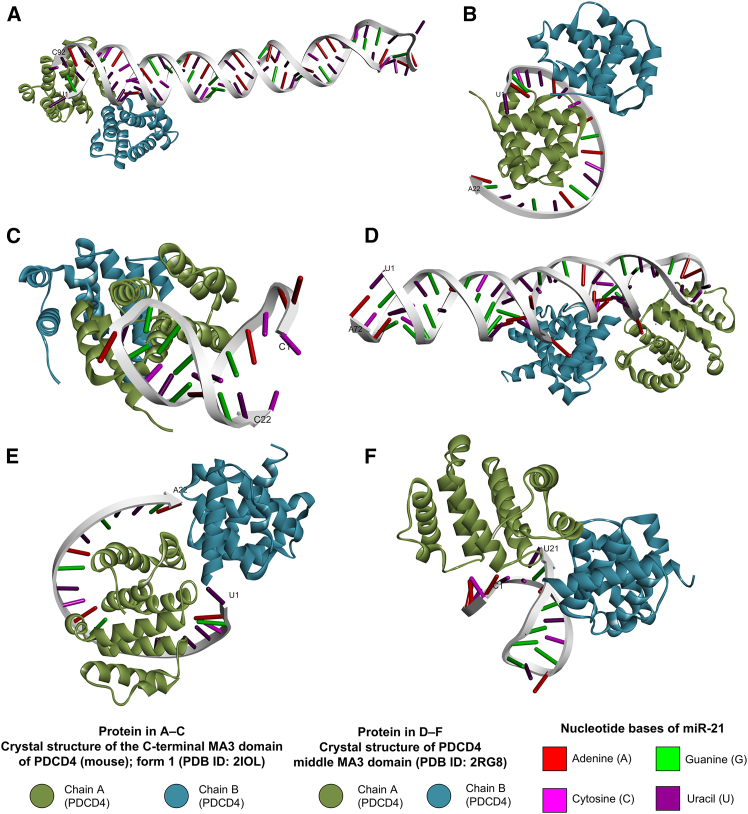
Predicted molecular docking interactions between miR-21 and mouse/human programmed cell death 4 (A) Stem-loop mmu-miR-21a and mouse PDCD4. (B) Mature mmu-miR-21a-5p and mouse PDCD4. (C) Mature mmu-miR-21a-3p and mouse PDCD4. (D) Stem-loop hsa-miR-21 and. Human PDCD4. (E) Mature hsa-miR-21-5p and human PDCD4. (F) Mature hsa-miR-21-3p and human PDCD4. Inset labels denote the names and numbers of the first and last nucleotide bases of miR-21. All figures were generated using BIOVIA Discovery Studio Visualizer.

Molecular docking simulations (Figures 6 and 7; Tables 1 and 2) demonstrated that miR-21 possesses strong binding affinities for both PTEN and PDCD4, supporting the biological plausibility of direct post-transcriptional regulation. Evaluation of the docking metrics indicated that lower (more negative) scores correspond to higher thermodynamic stability, while confidence scores exceeding 0.7 suggest a high probability of binding.

**Table 1:** Molecular docking simulation between PTEN/PDCD4 and miR-21

Protein	miRNA	Most negative docking score	Confidence score	Root mean squared deviation of RNA (Å)
PTEN (*Homo sapiens*)	stem-loop mmu-miR-21a	−334.39	0.9756	104.06
PTEN (*Homo sapiens*)	mature mmu-miR-21a-5p	−313.84	0.9636	87.57
PTEN (*Homo sapiens*)	mature mmu-miR-21a-3p	−305.02	0.9569	111.26
PTEN (*Homo sapiens*)	stem-loop hsa-miR-21	−304.80	0.9567	132.84
PTEN (*Homo sapiens*)	mature hsa-miR-21-5p	−313.84	0.9636	87.57
PTEN (*Homo sapiens*)	mature hsa-miR-21-3p	−251.04	0.8830	137.04
PDCD4 (*Mus musculus*)	stem-loop mmu-miR-21a	−240.61	0.8596	99.73
PDCD4 (*Mus musculus*)	mature mmu-miR-21a-5p	−334.74	0.9758	56.79
PDCD4 (*Mus musculus*)	mature mmu-miR-21a-3p	−247.33	0.8751	51.17
PDCD4 (*Homo sapiens*)	stem-loop hsa-miR-21	−273.55	0.9221	76.06
PDCD4 (*Homo sapiens*)	mature hsa-miR-21-5p	−298.77	0.9515	69.13
PDCD4 (*Homo sapiens*)	mature hsa-miR-21-3p	−251.04	0.8830	137.04

The docking simulations for PTEN indicated that mouse miR-21a has a higher binding affinity than human miR-21, substantiated by more negative docking score. The mmu-miR-21a stem-loop possessed the highest affinity, with a docking score of −334.39 and a confidence level of 0.9756. Comparative analysis of the mature mouse and human strands revealed that mmu-mid-21a-5p and hsa-miR-21-5p (docking scores: mouse miR-21a and human miR-21 = −313.84) have a more negative docking score than the 3p variants (docking scores: mouse miR-21a = −305.02 and human miR-21 = −251.04).

Molecular docking with the PDCD4 protein revealed that the mature mmu-miR-21a-5p exhibited the most robust interaction, yielding a docking score of −334.74 and a confidence score of 0.9758. hsa-miR-21-5p (docking score = −298.77) demonstrated a higher binding affinity compared with the 3p strand (docking score = −251.04). Similar trends were observed for murine sequences (docking score: mmu-miR-21a-3p = −247.33).

## Discussion

This study demonstrates that skipjack tuna bone-derived Bio serves as a potent dual regulator of bone remodeling by simultaneously enhancing osteoblast differentiation and attenuating RANKL-induced osteoclastogenesis. The dose-dependent increase in ALP activity and calcium deposition in MC3T3-E1 osteoblasts and C3H10T1/2 cells indicates that Bio effectively promotes the osteoblastogenesis. These observations align with recent paradigms, suggesting that marine-derived organic-complexes provide a superior substrate for hydroxyapatite crystallization compared with traditional inorganic salts.^10^^,^^11^^,^^12^^,^^19^^,^^20^ The presence of residual collagen peptides and essential trace elements within the Bio matrix likely facilitates this process by enhancing the bioavailability of calcium ions, thereby mimicking the natural bone microenvironment. Such synergy between the mineral phase and bioactive organic components has been previously noted to accelerate matrix maturation and strengthen bone architecture in preclinical models.^10^^,^^11^^,^^12^^,^^19^^,^^20^

On the resorptive side, Bio markedly suppressed the differentiation of RAW 264.7 macrophages into multinucleated osteoclasts, as evidenced by the reduction in TRAP activity and the smaller area of bone resorption pits. This inhibitory effect mirrors the action of established anti-resorptive agents and natural polyphenols that disrupt the RANKL/NF-κB axis.^2^^,^^21^^,^^22^ Mechanistically, the progression of osteoclastogenesis is heavily dependent on the accumulation of intracellular ROS, which serves as a second messenger to amplify RANKL signaling.^23^^,^^24^ The significant reduction in ROS production observed in this study suggests that Bio may exert an antioxidant-like effect during the osteoclast differentiation, similar to curcumin.^24^ This reduction in oxidative stress likely contributes to the observed downregulation of total NF-κB p65 expression, effectively dampening the inflammatory environment required for bone breakdowns.^25^^,^^26^

A major focus of this study is the characterization of the miR-21 signaling axis, which functions as a central epigenetic modulator in the skeletal microenvironment. miR-21 acts as a sophisticated molecular switch that facilitates bone resorption by post-transcriptionally silencing intrinsic inhibitors of osteoclast commitment.^15^ Our findings demonstrate that Bio treatment dose-dependently downregulates primary, precursor, and mature forms of miR-21, thereby relieving the suppression of its target genes, PTEN and PDCD4.^27^^,^^28^ Both targets are critical for limiting osteoclast maturation and maintaining bone homeostasis.^29^

Our findings demonstrate that Bio effectively counteracts osteoclastogenesis by reversing the miR-21/PTEN signaling axis in RANKL-stimulated RAW264.7 macrophages. Previous studies indicate that during standard osteoclast differentiation, elevated miR-21 levels typically suppress PTEN, thereby releasing the brake on downstream PI3K/AKT signaling to activate NFATc1. The biological outcome of this cascade has exhibited cell-type-specific divergence; while some studies show that miR-21 overexpression restricts PTEN and promotes AKT expression to favor bone progenitor regeneration and osteogenesis, contradictory evidence establishes that its upregulation in RANKL-stimulated RAW264.7 cells actively drives osteoclast maturation and TRAP activity. This bidirectional consequence hinges on the status of PTEN as a dual-affinity lipid phosphatase targeting phosphatidylinositol-3,4,5-triphosphate (PIP_3_), which sits at the crossroads of both skeletal degradation and mineral deposition. In inflammatory or RANKL-rich microenvironments, localized miR-21 spikes can trigger an osteoclastogenic cascade via canonical downstream transactivators like NF-κB and c-Fos.^30^ Our results show that Bio significantly upregulates PTEN gene expression in a concentration-dependent manner. This rescue of PTEN is further explained by the striking, dose-dependent suppression of the entire miR-21 biogenesis cascade—including pri-, pre-, and mature-miR-21 sequences—even within a PTEN-knockdown configuration. This confirms that Bio acts as a potent upstream inhibitor of miR-21 processing, restoring PTEN availability to suppress bone resorption.

To further isolate the functional interplay within this signaling loop, gain- and loss-of-function assays were conducted in a PTEN-deficient condition. When PTEN-knockdown cells were transfected with an miR-21 mimic to drive osteoclast commitment, subsequent treatment with higher concentrations of Bio forced an upregulation of relative NFATc1 levels, revealing that Bio may trigger alternative signaling mechanisms to regulate NFATc1 when the canonical PTEN pathway is disrupted. Conversely, when the upstream pathway was silenced using an miR-21 inhibitor, the addition of Bio operated synergistically, precipitating concentration-dependent decline in relative NFATc1 expression. Collectively, these findings confirm that Bio effectively blunts canonical osteoclastogenic pathways by suppressing upstream miR-21 biogenesis, which successfully preserves downstream target regulation even under compromised baseline conditions.

Notably, the Bio-mediated upregulation of these miR-21 targets was significantly enhanced in an osteoblast-osteoclast crosstalk environment. This synergistic response in OCM suggests that Bio may trigger or cooperate with the release of osteoblast-derived secretomes—also denoted as bone-derived factors—that work in concert to reinforce bone protection, particularly ALP.^31^ Molecular docking simulations further support the biological plausibility of this regulatory relationship, showing robust binding affinities and thermodynamic stability between miR-21 variants and the functional domains of PTEN and PDCD4.

**Figure 8:**
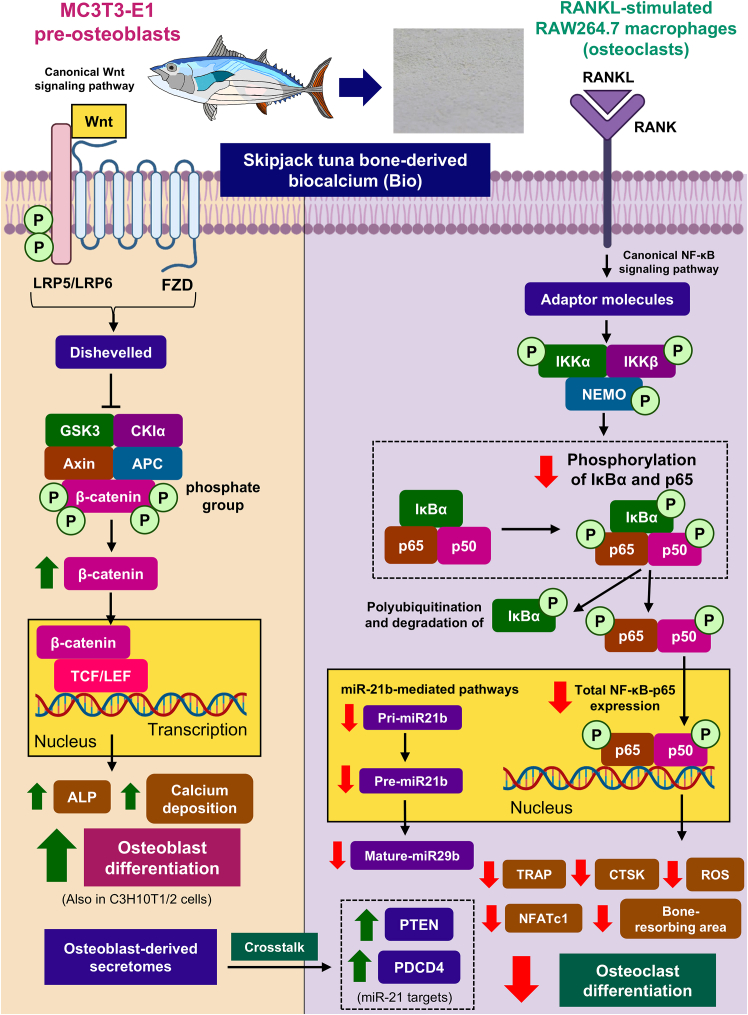
Proposed molecular mechanisms of skipjack tuna bone-derived Bio on the differentiation of mouse osteoblasts and osteoclasts and their intercellular crosstalk The circular icon with “P” represents a phosphate group. Sequential steps in signaling pathways are indicated by black arrows. The green upward and red downward arrows denote the upregulation and downregulation of specific biomolecules, respectively. ALP, alkaline phosphatase; APC, adenomatous polyposis coli; Bio, skipjack tuna bone-derived biocalcium; CK1 α, casein kinase 1 α; CTSK, Cathepsin K; FZD, frizzled receptor; GSK3, glycogen synthase kinase 3; IκBα, IκB inhibitor α; IKK, IκB kinase; LRP5/6, low-density lipoprotein receptor-related protein 5/6; miR, microRNA; NEMO, NF-κB essential modulator; NFATc1, nuclear factor of activated T cells, cytoplasmic 1; NF-κB, nuclear factor κ-light-chain-enhancer of activated B cells; PDCD4, programmed cell death 4; PTEN, phosphatase and tensin homolog; RANK, receptor activator of nuclear factor κB; RANKL, RANK ligand; ROS, reactive oxygen species; TCF/LEF, T cell factor/lymphoid enhancer-binding factor; TRAP, tartrate-resistant acid phosphatase.

From a translational perspective, the valorization of skipjack tuna bones into Bio addresses both nutritional needs and environmental concerns. By transforming a high-volume industrial byproduct into a bioactive supplement that simultaneously builds bone and inhibits its breakdown, this research supports the development of sustainable functional foods. The superior bioavailability and dual (osteoblastogenic and osteoclastogenic) regulatory actions (Figure 8) of Bio position it as a promising candidate for the prevention of osteoporosis in the aging population. In conclusion, skipjack tuna bone-derived Bio promotes skeletal health by modulating the ROS/miR-21 axis and enhancing intercellular communication, establishing a strong foundation for future clinical evaluation.

### Limitations of the study

Despite these promising cellular results, several limitations must be acknowledged. A primary technical constraint was that no phosphorylated-NF-κB-p65 bands were detected on western blot membranes. While total protein levels showed dose-dependent trends, particularly the suppression of total-NF-κB-p65 and the upregulation of β-catenin, definitive conclusions regarding the activation kinetics of these pathways remain preliminary without phosphorylation-specific data and cannot completely confirm definitive canonical pathway activation. This issue may stem from the transient nature of phosphorylation events^32^ or limitations in the sensitivity of our current detection equipment.

Additionally, this study exclusively focused on the miR-21 signaling axis based on its established significance in bone remodeling. However, Bio may simultaneously modulate other miRs or non-coding RNA profiles that contribute to osteoblastogenesis or osteoclastogenesis. The lack of a comprehensive high-throughput miR sequencing or microarray analysis limits our ability to rule out alternative pathways regulated by Bio.

Our investigations into the intercellular communication between osteoblasts and osteoclasts remain preliminary. Although we utilized OCM to assess the cell interactions, a detailed secretome profile—such as identifying specific exosomes, cytokines, or signaling proteins altered by Bio within the medium—was not performed. Consequently, the precise molecular cargo driving this cellular interplay warrants deeper proteomic characterization in future studies.

Furthermore, while MC3T3-E1, C3H10T1/2, and RAW 264.7 models are robust for mechanistic studies, they cannot fully replicate the systemic complexity of human bone remodeling, which involves hormonal regulation, mechanical loading, and gastrointestinal nutrient absorption. Future research should prioritize phosphorylation-specific analyses and utilize rodent models of osteoporosis, such as ovariectomized or SAMP8 mice, to validate these *in vitro* findings within a complex physiological environment.

## Resource availability

### Lead contact

Requests for further information and resources should be directed to and will be fulfilled by the lead contact, Yutthana Pengjam (yutthana.p@psu.ac.th).

### Materials availability

This study did not generate new unique reagents.

### Data and code availability

• All data reported in this paper will be shared by the lead contact upon request.
• This paper does not report original code.
• Any additional information required to reanalyze the data reported in this paper is available from the lead contact upon request.

## Acknowledgments

The authors gratefully acknowledge the financial support from the Program Management Unit for Competitiveness (PMUC), Thailand, under grant no. C02F670260, and the National Science, Research and Innovation Fund (NSRF) and 10.13039/501100004508Prince of Songkla University, under grant no. MET6901024S.

## Author contributions

Conceptualization, T.S., E.C., D.A., C.S.V., and Y.P.; methodology, S.J., T.S., S.P., S.K., P.W., J.S., J.K., E.C., D.A., C.S.V., and Y.P.; validation, S.J. and Y.P.; formal analysis, S.J., S.P., S.K., and Y.P.; investigation, S.J., T.S., S.P., S.K., P.W., J.S., J.K., E.C., D.A., C.S.V., and Y.P.; resources, T.S. and Y.P.; writing – original draft, S.J.; writing – review and editing, S.J. and Y.P.; visualization, S.J. and Y.P.; supervision, Y.P.; project administration, T.S., E.C., D.A., C.S.V., and Y.P.; and funding acquisition, T.S., E.C., D.A., C.S.V., and Y.P. All authors have read and agreed to the published version of the manuscript.

## Declaration of interests

The authors declare no competing interests.

## STAR★Methods

### Key resources table

**Table:** 

REAGENT or RESOURCE	SOURCE	IDENTIFIER
**Antibodies**		

Primary antibodies against β-catenin, total NF-κB p65, and β-actin	Cell Signaling Technology, USA	Cat# 9582; RRID: AB_823447; Cat# 3987; RRID: AB_2341215; Cat# 3700; RRID: AB_2242334
Horseradish peroxidase-conjugated secondary antibodies	Cell Signaling Technology, USA	Cat# 91196; RRID: AB_2940774; Cat# 7074; RRID: AB_2099233

**Biological samples**		

Skipjack tuna pre-cooked bones	Songkla Canning Public Co., Ltd., Songkhla, Thailand	N/A

**Chemicals, peptides, and recombinant proteins**		

α-Minimum Essential Medium (MEM) culture medium	Gibco Co., Bangkok, Thailand	3082975
RPMI 1640 culture medium	Gibco Co., Bangkok, Thailand	2902897
10% heat-inactivated fetal bovine serum	Gibco Co., Bangkok, Thailand	N/A
100× antibiotic–antimycotic	Gibco Co., Bangkok, Thailand	2913069
Recombinant Mouse TRANCE (RANKL) (carrier-free)	Advanced Medical Science, Bangkok, Thailand	N/A
MTT (3-(4,5-dimethylthiazol-2-yl)-2,5-diphenyltetrazolium bromide)	Thermo Fisher Scientific, Bangkok, Thailand	2311813
4% paraformaldehyde	Merck Co., Bangkok, Thailand	N/A
1% Alizarin red solution (ARS)	Merck Co., Bangkok, Thailand	4037011
Fluorescein-labeled calcium phosphate	PG Research Inc., Tokyo, Japan	N/A
Fast-Start SYBR Green Master Mix	Roche Diagnostics, Germany	N/A
OptiMEM reduced-serum medium	Invitrogen, NY, USA	N/A
HiPerfect transfection reagent	Qiagen, Valencia, CA, USA	N/A
RIPA buffer	Thermo Fisher Scientific, Inc., USA	N/A

**Critical commercial assays**		

Universal Mycoplasma Detection Kit	ATCC, Manassas, VA, USA	ATCC 30-1012K
ALP staining kit	Biodesign, Bangkok, Thailand	N/A
ALP assay kit	S.M. Chemical Supplies, Bangkok, Thailand	MAK447-1KT
TRAP staining kit	S.M. Chemical Supplies, Bangkok, Thailand	YBF-04
TRAP assay kit	S.M. Chemical Supplies, Bangkok, Thailand	L2503412C
OxiSelect intracellular ROS assay kit	CellBio Lab, Inc., CA, USA	N/A
miRNeasy kit	Qiagen, Valencia, CA, USA	N/A
miScript II RT kit	Qiagen, Valencia, CA, USA	N/A
SYBR Green PCR kit	Qiagen, Valencia, CA, USA	N/A

**Experimental models: Cell lines**		

Mouse (*Mus musculus*) MC3T3-E1 preosteoblasts	ATCC Cell Bank, Biomedia, Bangkok, Thailand	70070297
Mouse C3H10T1/2 cells	ATCC Cell Bank, Germany	N/A
Mouse RAW 264.7 macrophages	ATCC Cell Bank, Biomedia, Bangkok, Thailand	70066529

**Oligonucleotides**		

Primers: CTSK: Forward: 5′-ATGTGGGGGCTCAAGGTTCTG-3′ Reverse: 5′-CATATGGGAAAGCATCTTCAGAGTC-3′	Jantarawong et al.^22^	N/A
Primers: NFATc1: Forward: 5′-CCGTTGCTTCCAGAAAATAACA-3′ Reverse: 5′-TGTGGGATGTGAACTCGGAA-3′ PTEN: Forward: 5′-TGAAGACCATAACCCACCACA-3′ Reverse: 5′-TCATACACCAGTCCGTCCCT-3′ PDCD4: Forward: 5′-ACTGTGCCAACCAGTCCAAAGG-3′ Reverse: 5′-CCTCCACATCATACACCTGTCC-3′	Jantarawong et al.^21^, Liu et al.^33^, and Liang et al.^34^	N/A
Primers: GADPH: Forward: 5′-AAATGGTGAAGGTCGGTGTG-3′ Reverse: 5′-GAATTTGCCGTGAGTGGAGT-3′	Jantarawong et al.^22^, Liu et al.^33^, and Liang et al.^34^	N/A
PTEN small interfering RNA (siRNA)	Santa Cruz Biotechnology, Inc., TX, USA	N/A

**Software and algorithms**		

ImageJ software	https://imagej.net/ij/	Version 1.54g
RCSB PDB	https://www.rcsb.org/	accessed on February 17, 2026
miRBase database	https://www.mirbase.org/	accessed on February 17, 2026
RNAComposer server	https://rnacomposer.cs.put.poznan.pl/	accessed on February 18, 2026
HDOCK server	http://hdock.phys.hust.edu.cn/	accessed on February 18, 2026
BIOVIA Discovery Studio Visualizer	https://discover.3ds.com/discovery-studio-visualizer-download/	version 21.1.0.20298, accessed on February 19, 2026

**Other**		

Other reagents	Gibco Co., Bangkok, Thailand	N/A
Electric saw	Union Kitchen & Service, Thailand	W210E
High-pressure water jet	Andaman, Zinsano, Thailand	N/A
Milling machine	Yor Yong Hah Heng, Thailand	YCM-1.1E
Planetary ball mill	Retsch GmbH, Germany	PM 100
Multiskan FC microplate spectrophotometer	Thermo Scientific, Pittsburgh, PA, USA	N/A
Microscope integrated with a digital camera (ZEISS, Axiocam 705 color)	Bangkok, Thailand	N/A
Thermo Scientific Multiskan FC spectrophotometer	Pittsburgh PA, USA	N/A
Brightfield microscope (Olympus CX23)	Germany	N/A
Spectrofluorometer	Beckman Coulter, Inc., CA, USA	N/A
ABI Prism 7500 HT sequence detection system	Applied Biosystems, CA, USA	N/A
Chemiluminescence Vilber (Fusion Solo S) digital imaging system	Vilber, France	N/A

### Experimental model and study participant details

#### Bio preparation

The preparation Bio from skipjack tuna bones followed the protocols established in previous studies.^10^^,^^11^^,^^12^^,^^13^ Pre-cooked bones, sourced from skipjack tuna with an average weight of 6.5–7.5 kg, were provided by Songkla Canning Public Co., Ltd. (Songkhla, Thailand). These raw materials were transported in polyethylene bags to the Department of Food Technology at Prince of Songkla University within 1 h and maintained at −20°C for a duration not exceeding one month. For the initial pretreatment, an electric saw (W210E, Union Kitchen & Service, Thailand) was used to bisect the bones longitudinally. Subsequently, any remaining muscle tissue and impurities were eliminated using a high-pressure water jet (Andaman, Zinsano, Thailand) operated at 120 bar and a flow rate of 360 L/h for approximately 1–2 min.

To isolate the bone structure by removing non-collagenous proteins, the cleaned specimens were immersed in 2 M NaOH at a solid-to-liquid ratio of 1:10 (*w*/*v*). This alkaline treatment was performed at 50°C for 30 min with continuous agitation (150 rpm) using an overhead stirrer. Following the removal of the alkaline solution, the bones were rinsed under running water until the wash water reached a neutral or near-neutral pH. The samples were then briefly neutralized with 1.25 mM HCl, re-washed, and dried in a rotary tray dryer at 50°C for 2 h with an air velocity of 1.5 m/s. The dried bones were then reduced to a size of approximately 3–4 mm using a milling machine (YCM-1.1E, Yor Yong Hah Heng, Thailand).

The lipid content was removed from the granulated bones through a double extraction process using hexane (1:10 *w*/*v*) at 25°C for 60 min per cycle under constant stirring. After replacing the solvent once, the samples were allowed to dry at room temperature until the solvent odor was completely dissipated. To enhance the lightness and reduce the chromaticity of the defatted bones, a two-step bleaching process was implemented. The material was first treated with 2.5% (*v*/*v*) sodium hypochlorite for 30 min, followed by a second stage using 2.5% (*v*/*v*) hydrogen peroxide for 60 min, both under gentle stirring at ambient temperature. After thorough rinsing, the bleached bones were dried in a rotary tray dryer at 50°C for 5 h.

In the final stage, the dried material, designated as Bio, was subjected to fine pulverization in a planetary ball mill (PM 100, Retsch GmbH, Germany). This process utilized 25 stainless steel balls (20 mm diameter) at a rotational speed of 200 rpm for 2.5 h. The resulting powder was passed through a sieve to ensure a particle size of less than 75 μm. The final Biocalcium powder was then stored in airtight containers to prevent moisture absorption before further experimental analysis.

#### Cell culture

All cell lines used in this study were authenticated by the supplier (American Type Culture Collection; ATCC) before purchase. To maintain genetic stability and cell phenotypic integrity, all experiments were strictly performed using cells at low passage numbers (no more than passage 10) after thawing. All cell lines were screened and verified to be free of mycoplasma contamination using Universal Mycoplasma Detection Kit (ATCC 30-1012K, Manassas, VA, USA).

Mouse MC3T3-E1 pre-osteoblasts were cultured in α-MEM with supplements. Mouse RAW264.7 macrophages were cultured in RPMI 1640. Both media were enriched with 10% fetal bovine serum (FBS) and a 1% antibiotic–antimycotic solution. Both cell lines were incubated in a humidified incubator (5% CO_2_ and 37°C) until semiconfluent stage. For osteoblast differentiation, MC3T3-E1 cells were grown until they reached confluency in 6-well plates before being switched to an osteogenic induction medium. This specialized medium consisted of α-MEM supplemented with 50 μg/mL ascorbic acid, 5 mM β-glycerophosphate, and 100 nM dexamethasone.^35^^,^^36^ RAW264.7 macrophages were treated with 20 ng/mL RANKL and subsequently seeded into 6-, 24- or 96-well plates for 5 d^21^^,^^22^^,^^37^ to stimulate differentiation into osteoclasts. MC3T3-E1 osteoblasts and RANKL-stimulated RAW264.7 macrophages were treated with Bio dissolved in 1% ascorbic acid for further experiments. Untreated MC3T3-E1 osteoblasts and RANKL-stimulated RAW264.7 macrophages (no Bio) served as controls. The culture media were replenished every 3 d.

The mouse multipotent mesenchymal stem cell line C3H10T1/2 was cultured in Eagle’s basal medium supplemented with 10% heat-inactivated FBS as reported.^38^ The initial growth medium was further enriched with 4-(2-hydroxyethyl)-1-piperazineethanesulfonic acid (HEPES) buffer (pH 7.4), 10% FBS, and an antibiotic–antimycotic cocktail (penicillin, streptomycin, and amphotericin B). Cell culture was sustained within a humidified incubator under standard atmospheric conditions of 5% CO_2_ at 37°C until the semiconfluent state. For subsequent osteogenic differentiation, C3H10T1/2 cells were seeded into culture plates (8 ×10^4^ cells/well) and subsequently treated with Bio.

### Method details

#### Cell viability assay

Cell viability of MC3T3-E1 osteoblasts, RANKL-stimulated RAW 264.7 macrophages, and C3H10T1/2 cells were evaluated using the MTT colorimetric assay as reported.^36^^,^^39^ MC3T3-E1 osteoblasts and C3H10T1/2 cells (3×10^3^ cells/mL) were seeded into 96-well plates and permitted to adhere for 24 h before treatment. RAW 264.7 macrophages (5×10^3^ cells/mL) were seeded and subsequently exposed to 20 ng/mL of RANKL, concurrently with 0–30 μg/mL Bio for 5 d.

Following the respective treatment periods, 10 μL of MTT reagent was introduced to each well, and the plates were further incubated in a humidified environment for 4 h to allow the formation of formazan crystals. The culture supernatant was then meticulously removed and replaced with 100 μL of dimethyl sulfoxide to solubilize the dark blue crystals. After the plates were agitated on a microplate shaker for 10 min, the absorbance was measured at a wavelength of 570 nm using a microplate spectrophotometer (Multiskan FC, Thermo Scientific, Pittsburgh, PA, USA). The percentage of viable cells was determined by comparing the optical density of the treated samples against that of the untreated control groups.

#### ALP staining and activity

After 10 d of osteogenic induction with Bio, MC3T3-E1 osteoblasts and C3H10T1/2 cells were fixed and stained for ALP using ALP staining kit. Stained ALP-positive cells (dark purple) were captured using a microscope integrated with a digital camera (ZEISS, Axiocam 705 color, Thailand). For quantification, cell lysates were assayed for ALP activity using ALP assay kit by conversion of *p*-nitrophenyl phosphate to *p*-nitrophenol. Absorbance was measured at 405 nm using a spectrophotometer (Thermo Scientific Multiskan FC, Pittsburgh PA, USA). ALP activity was normalized to protein. ALP staining and assay were performed in triplicate (*n* = 3) and sextuplicate (*n* = 6), respectively.

#### Alizarin Red S (ARS) staining

ARS staining was performed to investigate calcium deposition (red staining) in MC3T3-E1 osteoblasts and C3H10T1/2 cells. MC3T3-E1 preosteoblasts and C3H10T1/2 cells were treated with Bio. After incubation for 14 d in a humidified chamber at 37°C, MC3T3-E1 osteoblasts and C3H10T1/2 cells were fixed in 4% paraformaldehyde (Merck Co., Bangkok, Thailand), and incubated in 1% ARS (Merck Co., Bangkok, Thailand) for 5 min at room temperature. Intracellular Ca^2+^ deposition was observed using a microscope integrated with a digital camera (ZEISS, Axiocam 705 color, Thailand). Quantitative mineralization was determined by measuring the absorbance at 550 nm using a spectrophotometer (Thermo Scientific Multiskan FC, Pittsburgh PA, USA). ARS staining and absorbance measurement were performed in triplicate (*n* = 3) and sextuplicate (*n* = 6), respectively.

#### TRAP staining and activity

RANKL-stimulated RAW264.7 macrophages treated were treated with Bio and incubated for 5 d. Then, cells were fixed and stained using TRAP staining kit. Images of TRAP-positive cells (red-stained cells) were captured using a microscope integrated with a digital camera (ZEISS, Axiocam 705 color, Thailand). For TRAP activity, cell lysates were assayed using TRAP assay kit and absorbance of liberated *p*-nitrophenol was measured at 405 nm using a spectrophotometer (Thermo Scientific Multiskan FC, Pittsburgh PA, USA). TRAP activity was normalized to protein and converted into percentage. TRAP staining and assay were performed in triplicate (*n* = 3).

#### *In vitro* bone resorption assay (bone pit assay)

An *in vitro* bone resorption assay was performed as reported^22^^,^^40^ to evaluate the bone-resorbing activity of differentiated osteoclasts. RANKL-stimulated RAW 264.7 macrophages (at a semiconfluent density of 1 × 10^3^ cells/mL) in 3 mL of supplemented RPMI 1640 medium were seeded onto 24-well bone resorption plates pre-coated with fluorescein-labeled calcium phosphate (PG Research Inc., Tokyo, Japan). Cells were treated with the highest non-cytotoxic concentrations of Bio and subsequently incubated at 37 °C in a humidified incubator with 5% CO_2_ for 21 d. After incubation, the wells were washed with phosphate-buffered saline (PBS), treated with 5% sodium hypochlorite for 5 min to remove remaining cells, rinsed with tap water, and air-dried. Resorption pits in each well were imaged using a microscope integrated with a digital camera (ZEISS, Axiocam 705 color, Thailand). The percentage of the resorbed area (pit area) was quantified using ImageJ software (version 1.54g). *In vitro* bone pit assay was performed in triplicate (*n* = 3).

#### Wright–Giemsa staining

RANKL-stimulated RAW 264.7 macrophages were treated with Bio in 6-well plates for 5 d. The cells were sedimented onto glass slides. The glass slides were air-dried, stained with Wright–Giemsa stain, and left to stand for 4 min. Then, phosphate buffer (pH 6.4) was dropped onto the slides, which were left to stand for 5 min, rinsed with distilled water, and dried. Images were captured using a brightfield microscope (Olympus CX23, Germany). Wright–Giemsa staining was performed in triplicate (*n* = 3).

#### Determination of intracellular ROS production

To evaluate the influence of Bio on the generation of intracellular ROS, an OxiSelect Intracellular ROS Assay Kit (CellBio Lab, Inc., CA, USA) was utilized. Initially, RANKL-stimulated murine RAW 264.7 macrophages were exposed to 0, 5, 10, and 20 μg/mL Bio for 12 h. Following this, the cells were labeled with 10 μM 2′,7′-dichlorofluorescein diacetate (DCFH-DA) to facilitate ROS detection. The resulting fluorescence intensity, which directly reflects the level of intracellular ROS accumulation, was monitored using a spectrofluorometer (Beckman Coulter, Inc., CA, USA). The measurements were conducted at specific excitation and emission wavelengths of 485 nm and 530 nm, respectively. The data were then expressed as a percentage of ROS production relative to the untreated control group to determine the antioxidant or regulatory effects of the treatment.

#### Western blot analysis

To quantify the protein levels of β-catenin and NF-κB p65, osteoclasts (4 × 10^5^ cells/well) were cultured in six-well plates. Following the respective treatments, total protein was isolated from cell lysates using RIPA buffer (Thermo Fisher Scientific, Inc., USA). The resulting lysates were prepared by centrifugation at 10,000 rpm for 10 min at 4°C, after which the supernatants were collected. Protein samples were subjected to SDS-PAGE using 12% polyacrylamide gels at a constant voltage of 100 V for 90 min. Subsequently, the resolved proteins were electroblotted onto polyvinylidene difluoride membranes at 100 V for 60 min. To prevent non-specific binding, the membranes were immersed in a blocking solution consisting of 5% non-fat dry milk in TBS-Tween for 4 h at room temperature. The membranes were then incubated overnight at 4°C with primary antibodies against β-catenin, total NF-κB p65, and β-actin (Cell Signaling Technology, USA), all diluted at a ratio of 1:1,000 in blocking buffer. After three 10-min washes with TBS-Tween, the membranes were treated with horseradish peroxidase-conjugated secondary antibodies (1:10,000 dilution; Cell Signaling Technology, USA) for 2 h at room temperature. Following a final washing step, protein bands were detected using an enhanced chemiluminescence substrate and visualized with the Chemiluminescence Vilber (Fusion Solo S) digital imaging system (Vilber, France). The relative intensity of the target bands was normalized against β-actin to determine changes in protein expression.

#### PTEN knockdown

To achieve silencing or knockdown of PTEN expression, transient transfection was performed using specific PTEN siRNA as reported.^38^ The introduction of siRNA into the osteoclasts was performed in accordance with the manufacturer's transfection guidelines. Following a 24-h post-transfection recovery period, the gene-silenced cells were subsequently subjected to Bio treatment for further downstream analysis.

#### Quantitative analysis of miRNA-21 and gene expression

To investigate the regulatory role of miR-21, miRNA-enriched total RNA was isolated from Bio-treated osteoclasts using the miRNeasy kit (Qiagen, Valencia, CA, USA) in accordance with the manufacturer’s instructions. The concentration and purity of the extracted RNA were determined, and 1 μg of total RNA was subsequently utilized for first-strand complementary DNA synthesis using the miScript II RT kit (Qiagen, Valencia, CA, USA). The quantification of the different transcriptional stages of miR-21, specifically the primary, precursor, and mature forms, was conducted through real-time PCR using the miScript Primer Assays in conjunction with a SYBR Green PCR kit (Qiagen, Valencia, CA, USA). These analyses were performed on an ABI Prism 7500 HT sequence detection system (Applied Biosystems, CA, USA). In parallel, the mRNA expression of CTSK and NFATc1, key markers of osteoclast activity, was evaluated. Total RNA was reverse-transcribed, and the resulting complementary DNA was amplified using specific primers and Fast-Start SYBR Green Master Mix (Roche Diagnostics, Germany). The relative expression levels of both miR-21 variants and the CTSK and NFATc1 genes were normalized against GAPDH and calculated using the 2^−ΔΔCt^ method.

#### miRNA transfection and functional assays

The functional involvement of miR-21 in cellular processes was further elucidated through transient transfection experiments. Either specific miR-21 mimic or inhibitor was introduced into Bio-treated osteoclasts to effectively silence the target miRNA. The transfection process was carried out in OptiMEM reduced-serum medium (Invitrogen, NY, USA) using the HiPerfect transfection reagent (Qiagen, Valencia, CA, USA). After a 48-hour incubation period, the transfected cells were harvested for downstream functional analyses. The efficacy of the miR-21 knockdown was assessed by monitoring changes in the expression of CTSK. Furthermore, the impact on osteoclast function was quantified by measuring the percentage of TRAP activity.

#### Crosstalk between osteoblasts and osteoclasts

To investigate the intercellular communication between osteoblasts and osteoclasts, osteoblast-conditioned medium (OCM) was first prepared from MC3T3-E1 osteoblasts. These osteoblasts were cultured in an osteogenic induction medium for a specified duration to allow for the secretion of bioactive factors into the supernatant. After the OCM was harvested and filtered, murine RAW 264.7 macrophages were seeded and stimulated with 20 ng/mL of RANKL to initiate their differentiation into osteoclasts. The cells were then maintained under two distinct experimental environments: (1) standard RANKL-supplemented differentiation medium (supplemented RPMI treated with 20 ng/mL of RANKL) and (2) standard medium enriched with 10% (*v*/*v*) OCM to simulate the osteoblast-osteoclast crosstalk environment. Within these groups, the differentiating osteoclasts were concurrently exposed to skipjack tuna bone-derived Bio at concentrations of 2.5, 5, 10, and 20 μg/mL. Following the incubation period, the regulatory impact of Bio and the osteoblast-derived secretomes was assessed by analyzing the expression levels of the target genes PTEN and PDCD4. The method of qRT-PCR similar to the measurement of gene expression as mentioned in section “Quantitative Analysis of miRNA-21 and Gene Expression” was applied.

#### Computational modeling and molecular docking simulation

To evaluate the potential for interactions between miR-21 and its downstream targets, molecular docking simulations were conducted. The three-dimensional structures of the human PTEN protein (Protein Data Bank [PDB]: 1D5R), mouse PDCD4 protein (PDB: 2IOL), and human PDCD4 protein (PDB: 2RG8) were retrieved from the RCSB PDB^41^ (https://www.rcsb.org/; accessed on February 17, 2026) in PDB format. The stem-loop (precursor) and mature sequences for both mouse (*Mus musculus*, mmu-) and human (*Homo sapiens*, hsa-) miR-21 were obtained from the miRBase database^42^ (https://www.mirbase.org/; accessed on February 17, 2026, Table 2).

**Table 2:** Sequences of the stem-loop and mature forms of mmu- and hsa-miR-21

miRNA	Accession	Sequence
Stem-loop mmu-miR-21a (mmu-pre-miR-21a)^a^	MI0000569	UGUACCACCUUGUCGGAUAGCUUAUCAGACUGAUGUUGACUGUUGAAUCUCAUGGCAACAGCAGUCGAUGGGCUGUCUGACAUUUUGGUAUC
Mature mmu-miR-21a-5p	MIMAT0000530	18-UAGCUUAUCAGACUGAUGUUGA-39
Mature mmu-miR-21a-3p	MIMAT0004628	56-CAACAGCAGUCGAUGGGCUGUC-77
Stem-loop hsa-miR-21 (hsa-pre-miR-21)^b^	MI0000077	UGUCGGGUAGCUUAUCAGACUGAUGUUGACUGUUGAAUCUCAUGGCAACACCAGUCGAUGGGCUGUCUGACA
Mature hsa-miR-21-5p	MIMAT0000076	8-UAGCUUAUCAGACUGAUGUUGA-29
Mature hsa-miR-21-3p	MIMAT0004494	46-CAACACCAGUCGAUGGGCUGU-66

a mmu, *Mus musculus*.

b hsa, *Homo sapiens*.

Before docking, the sequence homology between the human PTEN and its corresponding *M. musculus* ortholog was verified using BLASTP, following the parameters described by Jantarawong et al.^39^ Sequence alignment confirmed that the identity between human and mouse PTEN significantly exceeded the 30% threshold (93.06%), indicating high structural conservation and validating the use of human protein structure for predicting molecular interactions in a mouse cell model.^39^

The three-dimensional RNA structures of the miR-21 variants was predicted using the RNAComposer server^43^ (https://rnacomposer.cs.put.poznan.pl/, accessed on February 18, 2026) and retrieved as PDB files. Subsequent docking procedures were performed on the HDOCK server^44^ (http://hdock.phys.hust.edu.cn/, accessed on February 18, 2026), to analyze binding affinities between the protein targets and the RNA ligands (human PTEN and mmu- and hsa-miR21, mouse PTEN and mmu-miR-21, and human PTEN and hsa-miR-21). The resulting computational models were evaluated based on docking energy scores and confidence levels. All molecular interaction visualizations were generated using BIOVIA Discovery Studio Visualizer (version 21.1.0.20298; https://discover.3ds.com/discovery-studio-visualizer-download/, accessed on February 19, 2026).

### Quantification and statistical analysis

All data are presented as mean ± standard deviation (SD) and were derived from experiments conducted in triplicate or with 6 to 8 replicates, depending on the assay. Effect sizes were calculated using Cohen’s *d* to assess the magnitude of differences between experimental and control groups. The normality of data distribution within each group was evaluated using the Shapiro–Wilk test. For datasets where all groups followed a normal distribution, differences between two groups (e.g., treated vs. control) were assessed using a Student’s t-test. If at least one group in a given dataset did not meet the assumption of normality, group comparisons were made using the non-parametric Mann–Whitney U test (one-tailed). Statistical significance was defined as *p* < 0.05, and significant differences were indicated with an asterisk (∗) in all relevant bar graphs.
