# Supplementary material for: Skipjack tuna bone-derived biocalcium ameliorates osteoblast and osteoclast differentiation through microRNA21 regulation
Source: iScience. 2026 Jul 16;29(8):116808. doi: 10.1016/j.isci.2026.116808 (PMC13400949; doi:10.1016/j.isci.2026.116808)
Supplement: Document S1. Figures S1–S12 and Tables S1–S82 [file mmc1.pdf]

iScience, Volume *29*

Supplemental information

Skipjack tuna bone-derived biocalcium ameliorates osteoblast and osteoclast differentiation through microRNA21 regulation

Sompot Jantarawong, Sudarat Phuntong, Saowapak Kanobthammakul, Papitchaya Watcharanurak, Jidapa Szekely, Jedsada Kaewrakmuk, Eakolarn Chotianuson, Doungporn Amornlerdpison, Chutima S. Vaddhanaphuti, Theeraphol Senphan, and Yutthana Pengjam

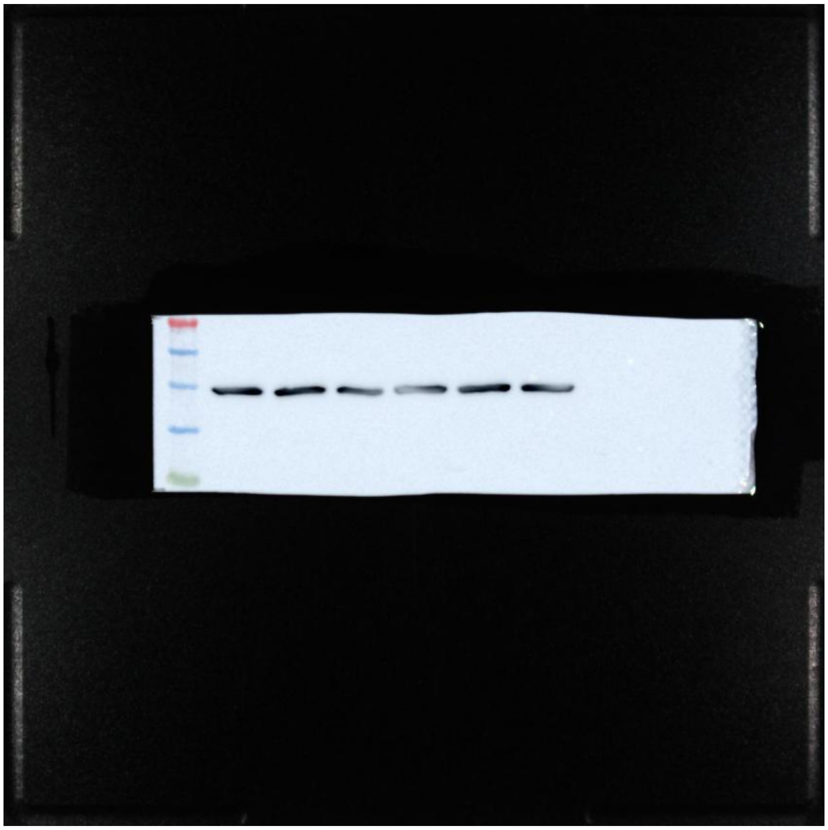

Figure S1. Original Western blot image of β-actin in MC3T3-E1 osteoblasts (replicate 1).

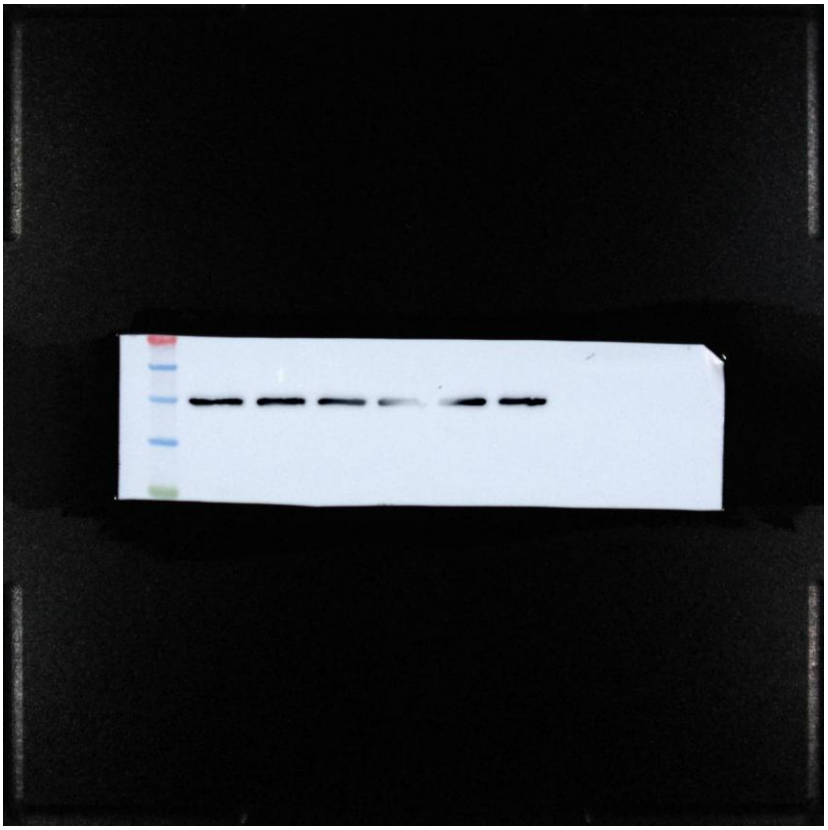

Figure S2. Original Western blot image of β-actin in MC3T3-E1 osteoblasts (replicate 2).

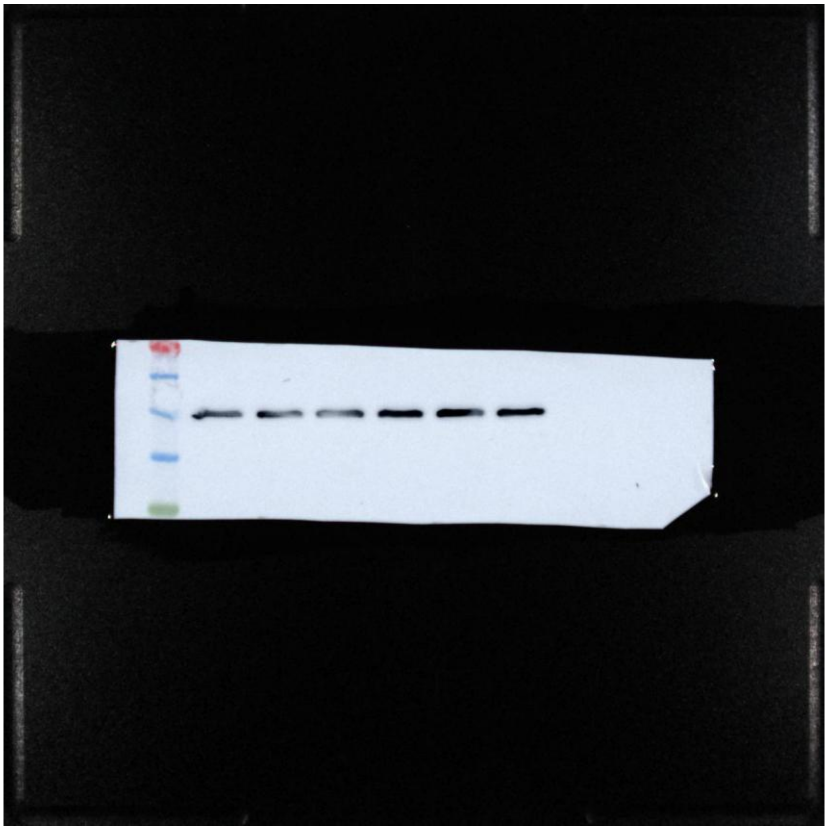

Figure S3. Original Western blot image of β-actin in MC3T3-E1 osteoblasts (replicate 3).

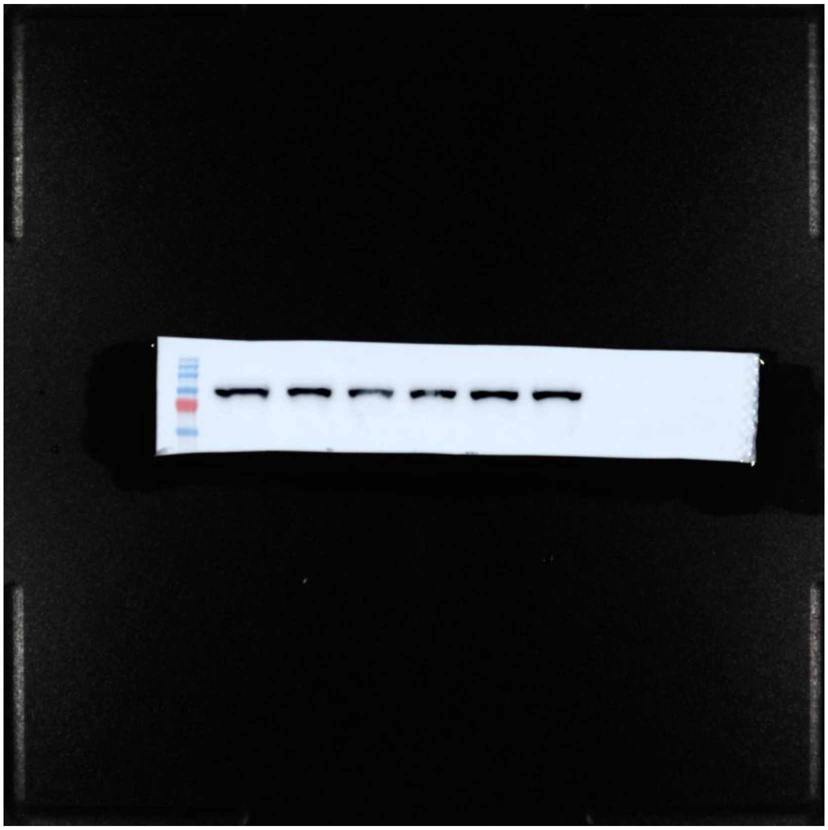

Figure S4. Original Western blot image of β-catenin in MC3T3-E1 osteoblasts (replicate 1).

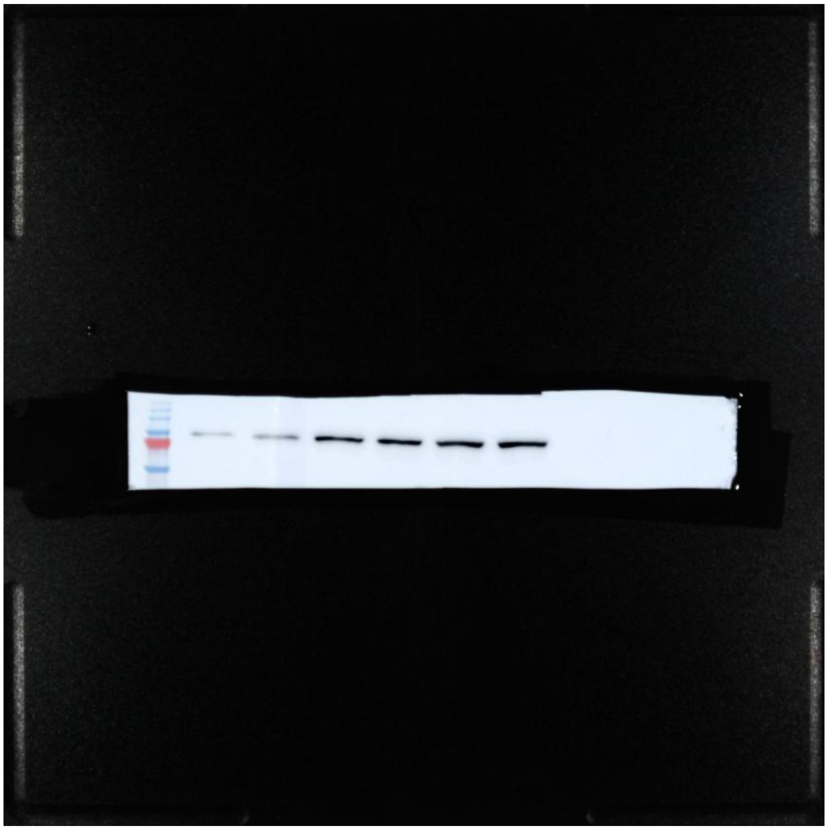

Figure S5. Original Western blot image of β-catenin in MC3T3-E1 osteoblasts (replicate 2).

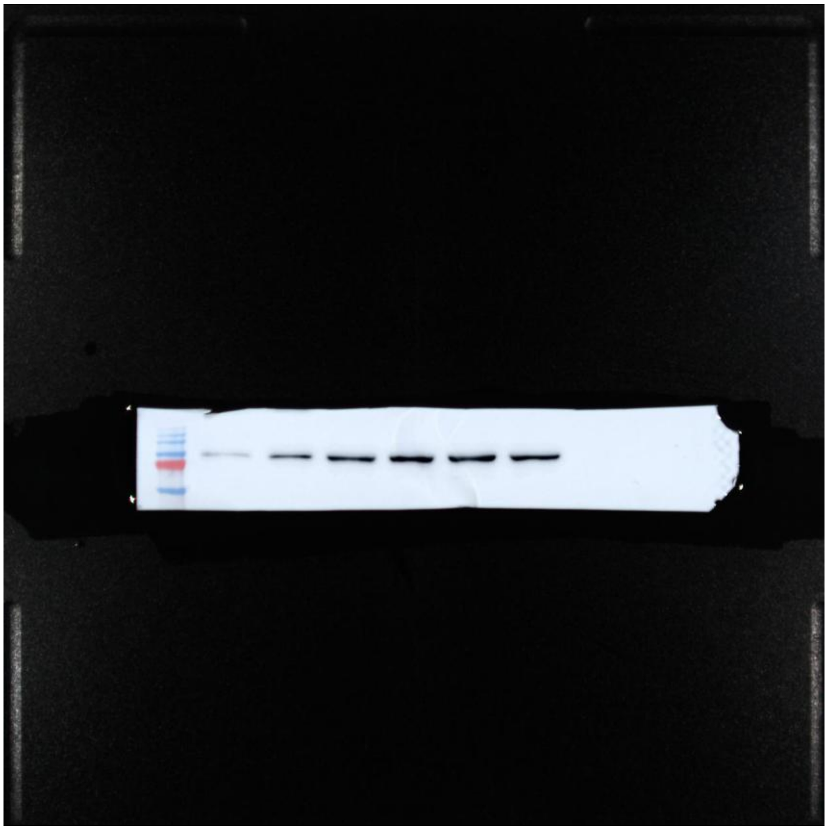

Figure S6. Original Western blot image of β-catenin in MC3T3-E1 osteoblasts (replicate 3).

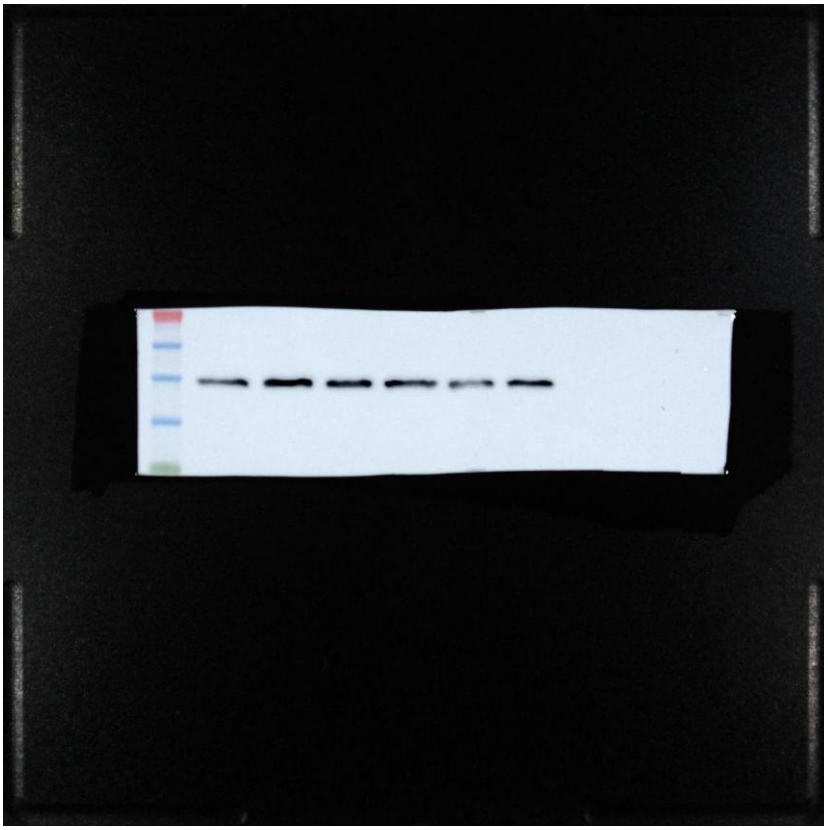

Figure S7. Original Western blot image of β-actin in osteoclasts (replicate 1).

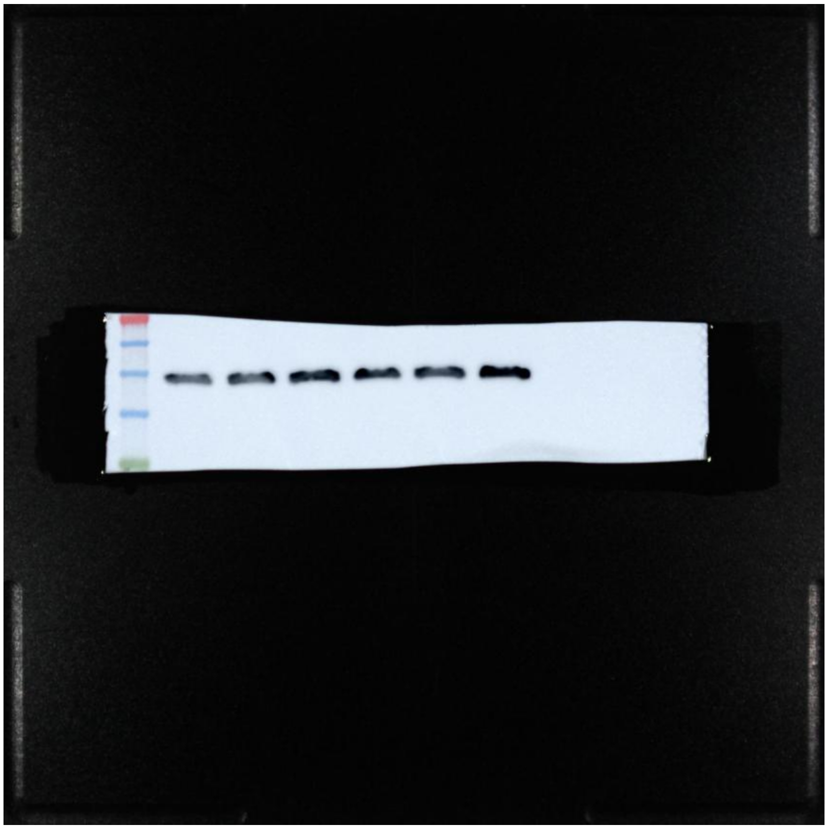

Figure S8. Original Western blot image of β-actin in osteoclasts (replicate 2).

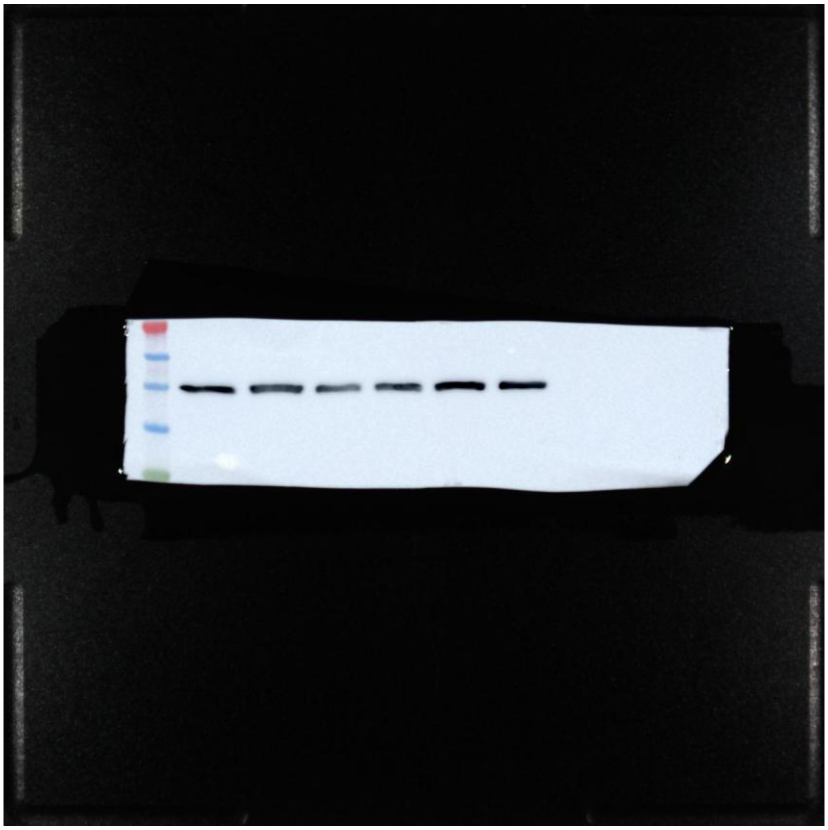

Figure S9. Original Western blot image of β-actin in osteoclasts (replicate 3).

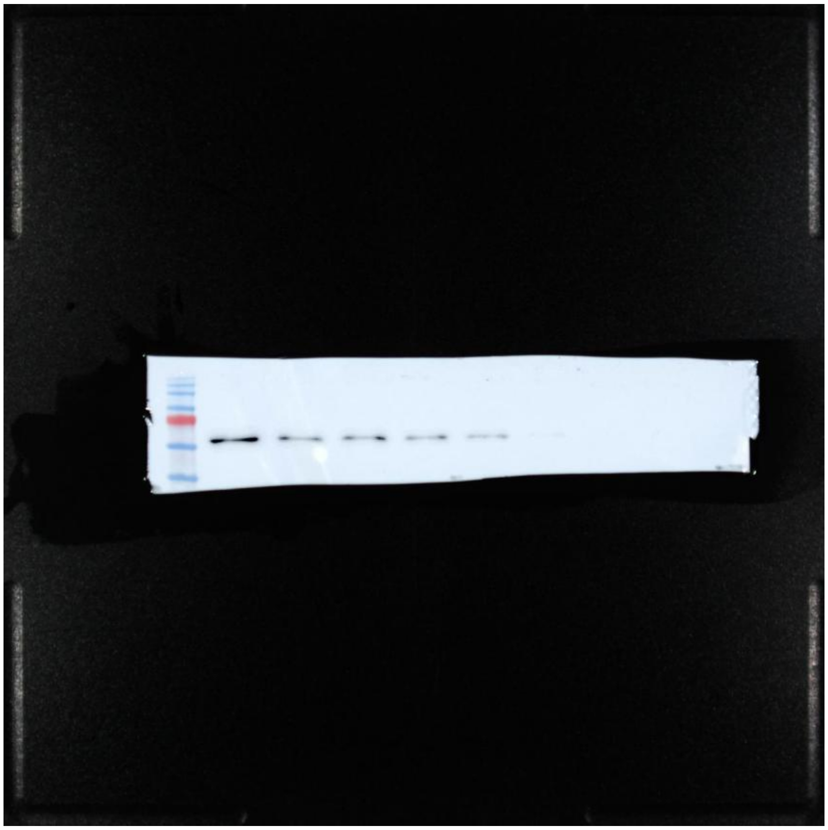

Figure S10. Original Western blot image of NF-κB p65 in osteoclasts (replicate 1).

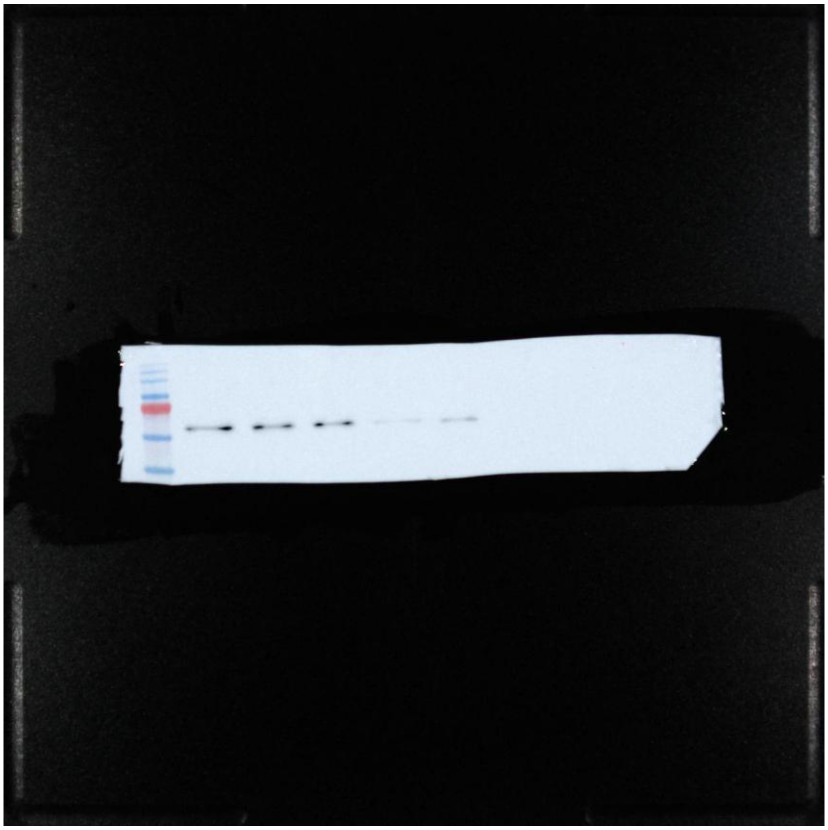

Figure S11. Original Western blot image of NF-κB p65 in osteoclasts (replicate 2).

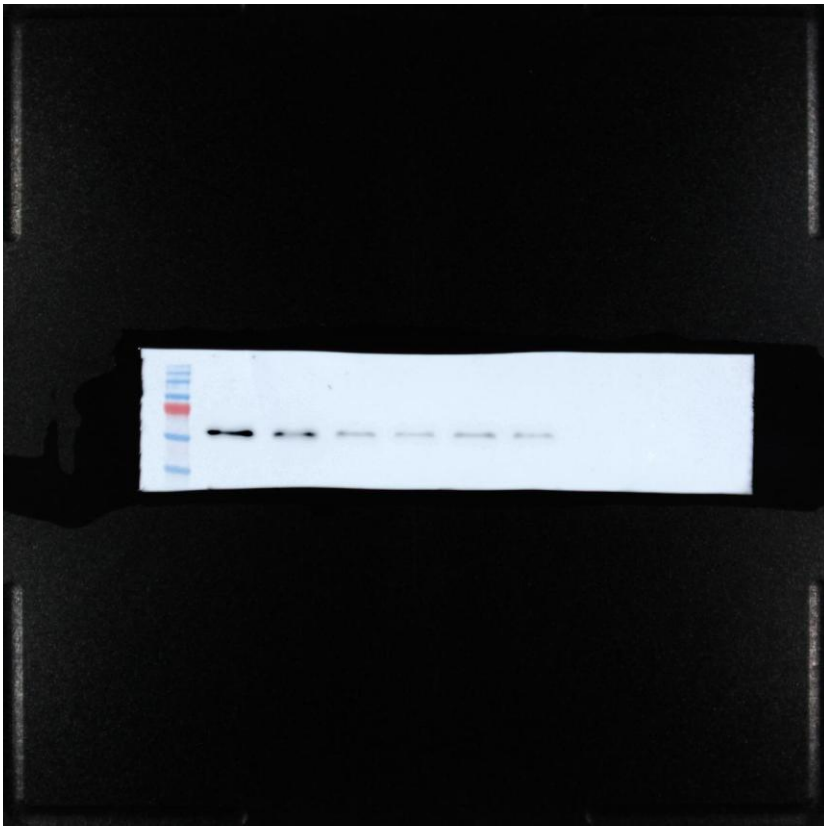

Figure S12. Original Western blot image of NF-κB p65 in osteoclasts (replicate 3).

Table S1: Descriptive statistics and Cohen's *d* of absorbance at 570 nm in MTT assay in MC3T3-E1 osteoblasts

Sample	MC3T3-E1	Bio1	Bio2.5	Bio5	Bio10	Bio20	Bio30
Average	0.4518	0.4414	0.4385	0.4388	0.4370	0.3919	0.1205
Standard deviation	0.0146	0.0362	0.0316	0.0340	0.0315	0.0333	0.0203
Margin of error for a 95% confidence interval	0.0122	0.0303	0.0265	0.0285	0.0263	0.0278	0.0170
Lower bound of 95% confidence interval	0.4397	0.4111	0.4121	0.4103	0.4106	0.3640	0.1035
Upper bound of 95% confidence interval	0.4640	0.4717	0.4650	0.4673	0.4633	0.4197	0.1375
Cohen's *d* (compared with MC3T3-E1)	Not applicable	−0.3780	−0.5409	−0.4983	−0.6061	−2.3347	−18.7354

Table S2: Shapiro–Wilk test of absorbance at 570 nm in MTT assay in MC3T3-E1 osteoblasts

Sample	MC3T3-E1	Bio1	Bio2.5	Bio5	Bio10	Bio20	Bio30
p-value	0.6279	0.2628	0.7956	0.8595	0.6349	0.7873	0.7521
W	0.9349	0.8919	0.95	0.9562	0.9355	0.9492	0.946
B	0.03728	0.09053	0.0816	0.08808	0.08061	0.08578	0.05231
Skewness	0.925	−0.06005	0.4328	−0.1393	0.4404	0.6798	0.3394
Skewness shape	Potentially symmetrical (*p*-value = 0.219)	Potentially symmetrical (*p*-value = 0.936)	Potentially symmetrical (*p*-value = 0.565)	Potentially symmetrical (*p*-value = 0.853)	Potentially symmetrical (*p*-value = 0.558)	Potentially symmetrical (*p*-value = 0.366)	Potentially symmetrical (*p*-value = 0.652)
Excess kurtosis	0.5877	−1.8616	−1.0598	−1.2459	−0.1945	−0.1876	−1.1921
Kurtosis shape	Potentially Mesokurtic, normal like tails (*p*-value = 0.691)	Potentially Mesokurtic, normal like tails (*p*-value = 0.209)	Potentially Mesokurtic, normal like tails (*p*-value = 0.474)	Potentially Mesokurtic, normal like tails (*p*-value = 0.4)	Potentially Mesokurtic, normal like tails (*p*-value = 0.895)	Potentially Mesokurtic, normal like tails (*p*-value = 0.899)	Potentially Mesokurtic, normal like tails (*p*-value = 0.421)
Outliers	No outliers	No outliers	No outliers	No outliers	No outliers	No outliers	No outliers

Table S3: One-way analysis of variance of absorbance at 570 nm in MTT assay in MC3T3-E1 osteoblasts

Source of variation	Sum of square	Degree of freedom	Mean square	F statistic	*p*-value	F critical value
Between groups	0.6884	6	0.1147	129.5634	2.6104×10^{-28}	2.2904
Within groups	0.0434	49	0.0009			
Total	0.7317	55				

Table S4: Descriptive statistics and Cohen's *d* of ALP activity (mmol/μg protein) in MC3T3-E1 osteoblasts

Sample	MC3T3-E1	Bio2.5	Bio5	Bio10	Bio20
Average	1.0320	1.2615	1.4480	1.7158	2.0955
Standard deviation	0.1053	0.0458	0.1109	0.1413	0.1172
Margin of error for a 95% confidence interval	0.1105	0.0481	0.1163	0.1483	0.1230
Lower bound of 95% confidence interval	0.9215	1.2134	1.3317	1.5676	1.9725
Upper bound of 95% confidence interval	1.1425	1.3096	1.5643	1.8641	2.2185
Cohen's *d* (compared with MC3T3-E1)	Not applicable	2.8259	3.8476	5.4883	9.5436

Table S5: Shapiro–Wilk test of ALP activity (mmol/μg protein) in MC3T3-E1 osteoblasts

Sample	MC3T3-E1	Bio2.5	Bio5	Bio10	Bio20
p-value	0.6832	0.3172	0.8015	0.1995	0.4133
W	0.9291	0.8816	0.9419	0.8556	0.8968
B	0.227	0.09622	0.2406	0.2922	0.2483
Skewness	0.696	0.6834	−0.04448	0.7267	0.3918
Skewness shape	Potentially symmetrical (*p*-value = 0.41)	Potentially symmetrical (*p*-value = 0.419)	Potentially symmetrical (*p*-value = 0.958)	Potentially symmetrical (*p*-value = 0.39)	Potentially symmetrical (*p*-value = 0.643)
Excess kurtosis	−0.8464	−1.477	−1.3343	−1.688	−2.0756
Kurtosis shape	Potentially Mesokurtic, normal like tails (*p*-value = 0.627)	Potentially Mesokurtic, normal like tails (*p*-value = 0.396)	Potentially Mesokurtic, normal like tails (*p*-value = 0.443)	Potentially Mesokurtic, normal like tails (*p*-value = 0.332)	Potentially Mesokurtic, normal like tails (*p*-value = 0.233)
Outliers	No outliers	No outliers	No outliers	No outliers	No outliers

Table S6: One-way analysis of variance of ALP activity (mmol/μg protein) in MC3T3-E1 osteoblasts

Source of variation	Sum of square	Degree of freedom	Mean square	F statistic	*p*-value	F critical value
Between groups	4.0755	4	1.0189	86.0777	3.0484×10^{-14}	2.7587
Within groups	0.2959	25	0.0118			
Total	4.3715	29				

Table S7: Descriptive statistics and Cohen's *d* of absorbance at 550 nm in Alizarin red S staining in MC3T3-E1 osteoblasts

Sample	MC3T3-E1	Bio2.5	Bio5	Bio10	Bio20
Average	0.0527	0.0798	0.1156	1.4133	1.6060
Standard deviation	0.0068	0.0097	0.0097	0.0839	0.0713
Margin of error for a 95% confidence interval	0.0072	0.0102	0.0101	0.0881	0.0748
Lower bound of 95% confidence interval	0.0455	0.0696	0.1054	1.3253	1.5312
Upper bound of 95% confidence interval	0.0598	0.0900	0.1257	1.5014	1.6808
Cohen's *d* (compared with MC3T3-E1)	Not applicable	3.2305	7.5194	22.8576	30.6619

Table S8: Shapiro–Wilk test of absorbance at 550 nm in Alizarin red S staining in MC3T3-E1 osteoblasts

Sample	MC3T3-E1	Bio2.5	Bio5	Bio10	Bio20
p-value	0.9862	0.05956	0.3858	0.1433	0.7896
W	0.972	0.7878	0.8928	0.8373	0.9406
B	0.01503	0.01932	0.02042	0.1717	0.1547
Skewness	−0.2901	0.08855	−1.1184	−0.5398	0.5219
Skewness shape	Potentially symmetrical (*p*-value = 0.731)	Potentially symmetrical (*p*-value = 0.917)	Potentially symmetrical (*p*-value = 0.186)	Potentially symmetrical (*p*-value = 0.523)	Potentially symmetrical (*p*-value = 0.537)
Excess kurtosis	−0.2867	−3.0465	0.8147	−2.1117	1.1817
Kurtosis Shape	Potentially Mesokurtic, normal like tails (*p*-value = 0.869)	Potentially Mesokurtic, normal like tails (*p*-value = 0.08)	Potentially Mesokurtic, normal like tails (*p*-value = 0.64)	Potentially Mesokurtic, normal like tails (*p*-value = 0.225)	Potentially Mesokurtic, normal like tails (*p*-value = 0.497)
Outliers	No outliers	No outliers	No outliers	No outliers	1.723

Table S9: One-way analysis of variance of absorbance at 550 nm in Alizarin red S staining in MC3T3-E1 osteoblasts

Source of variation	Sum of square	Degree of freedom	Mean square	F statistic	*p*-value	F critical value
Between groups	14.7846	4	3.6962	1495.0038	2.3522×10^{-29}	2.7587
Within groups	0.0618	25	0.0025			
Total	14.8464	29				

Table S10: Descriptive statistics and Cohen's *d* of the relative expression of β-catenin (normalized with β-actin) in MC3T3-E1 osteoblasts

Sample	Bio1	Bio2.5	Bio5	Bio10	Bio20
Average	1.0000	5.1301	14.5732	6.0120	8.1560
Standard deviation	0.0000	2.9555	3.9555	4.9555	5.9555
Margin of error for a 95% confidence interval	26.5538	35.5385	44.5231	53.5078	62.4924
Lower bound of 95% confidence interval	−21.4238	−20.9653	−38.5111	−45.3517	−50.6681
Upper bound of 95% confidence interval	31.6839	50.1117	50.5351	61.6638	74.3167
Cohen's *d* (compared with OC)	1.2083	1.6132	0.9679	1.5026	1.5173

Table S11: Shapiro–Wilk test of the relative expression of β-catenin (normalized with β-actin) in MC3T3-E1 osteoblasts

Sample	Bio1	Bio2.5	Bio5	Bio10	Bio20
p-value	1	1	1	1	1
W	1	1	1	1	1
B	2.9554	0.04378	6.6554	1.2352	3.586

Table S12: One-way analysis of variance of the relative expression of β-catenin (normalized with β-actin) in MC3T3-E1 osteoblasts

Source of variation	Sum of square	Degree of freedom	Mean square	F statistic	*p*-value	F critical value
Between groups	279.9889	5	55.9978	5.8143	0.0196	3.9715
Within groups	67.4177	7	9.6311			
Total	347.4066	12				

Table S13: Descriptive statistics and Cohen's *d* of absorbance at 570 nm in MTT assay in C3H10T1/2 cells

Sample	C3H10T1/2	Bio1	Bio2.5	Bio5	Bio10	Bio20	Bio30
Average	0.4847	0.4881	0.4570	0.4144	0.4110	0.4340	0.1097
Standard deviation	0.0383	0.0524	0.0439	0.0407	0.0554	0.0507	0.0191
Margin of error for a 95% confidence interval	0.0320	0.0438	0.0367	0.0341	0.0463	0.0424	0.0160
Lower bound of 95% confidence interval	0.4526	0.4443	0.4203	0.3803	0.3646	0.3916	0.0937
Upper bound of 95% confidence interval	0.5167	0.5319	0.4937	0.4485	0.4573	0.4764	0.1256
Cohen's *d* (compared with C3H10T1/2)	Not applicable	0.0746	−0.6715	−1.7765	−1.5475	−1.1283	−12.3867

Table S14: Shapiro–Wilk test of absorbance at 570 nm in MTT assay in C3H10T1/2 cells

Sample	C3H10T1/2	Bio1	Bio2.5	Bio5	Bio10	Bio20	Bio30
p-value	0.4774	0.05045	0.2929	0.2059	0.006368	0.05207	0.07749
W	0.9203	0.8162	0.8969	0.8807	0.712	0.8177	0.8362
B	0.09726	0.1252	0.11	0.1012	0.1237	0.1212	0.04621
Skewness	−0.6965	−1.2361	−0.1392	1.402	2.2861	1.0333	−0.03271
Skewness shape	Potentially symmetrical (*p*-value = 0.354)	Potentially symmetrical (*p*-value = 0.1)	Potentially symmetrical (*p*-value = 0.853)	Potentially symmetrical (*p*-value = 0.062)	Potentially symmetrical (*p*-value = 0.002)	Potentially symmetrical (*p*-value = 0.169)	Potentially symmetrical (*p*-value = 0.965)
Excess kurtosis	−0.8743	0.2259	−1.9971	3.1438	6.0262	−0.4319	−2.4032
Kurtosis shape	Potentially Mesokurtic, normal like tails (*p*-value = 0.555)	Potentially Mesokurtic, normal like tails (*p*-value = 0.879)	Potentially Mesokurtic, normal like tails (*p*-value = 0.177)	Potentially Mesokurtic, normal like tails (*p*-value = 0.034)	Potentially Mesokurtic, normal like tails (*p*-value = 0.0)	Potentially Mesokurtic, normal like tails (*p*-value = 0.771)	Potentially Mesokurtic, normal like tails (*p*-value = 0.105)
Outliers	No outliers	No outliers	No outliers	0.5012	0.5416	No outliers	No outliers

Table S15: Descriptive statistics and Cohen's *d* of ALP activity (mmol/µg protein) in C3H10T1/2 cells

Sample	C3H10T1/2	Bio2.5	Bio5	Bio10	Bio20
Average	0.5023	0.7856	1.0004	1.3887	2.0073
Standard deviation	0.1005	0.0595	0.0705	0.1378	0.1629
Margin of error for a 95% confidence interval	0.0929	0.0550	0.0652	0.1275	0.1507
Lower bound of 95% confidence interval	0.4093	0.7306	0.9352	1.2612	1.8566
Upper bound of 95% confidence interval	0.5952	0.8406	1.0656	1.5162	2.1579
Cohen's *d* (compared with C3H10T1/2)	Not applicable	3.4304	5.7383	7.3491	11.1197

Table S16: Shapiro–Wilk test of ALP activity (mmol/µg protein) in C3H10T1/2 cells

Sample	C3H10T1/2	Bio2.5	Bio5	Bio10	Bio20
p-value	0.1822	0.7005	0.8629	0.6869	0.6541
W	0.8641	0.9367	0.9532	0.9354	0.9322
B	0.2288	0.141	0.1686	0.3265	0.3853
Skewness	−1.1348	0.08086	0.8065	0.5635	1.0074
Skewness shape	Potentially symmetrical (*p*-value = 0.153)	Potentially symmetrical (*p*-value = 0.919)	Potentially symmetrical (*p*-value = 0.31)	Potentially symmetrical (*p*-value = 0.478)	Potentially symmetrical (*p*-value = 0.204)
Excess kurtosis	0.2408	-0.4693	0.5683	-0.527	0.9969
Kurtosis shape	Potentially Mesokurtic, normal like tails (*p*-value = 0.879)	Potentially Mesokurtic, normal like tails (*p*-value = 0.767)	Potentially Mesokurtic, normal like tails (*p*-value = 0.72)	Potentially Mesokurtic, normal like tails (*p*-value = 0.74)	Potentially Mesokurtic, normal like tails (*p*-value = 0.53)
Outliers	No outliers	No outliers	No outliers	No outliers	2.305

Table S17: One-way analysis of variance of ALP activity (mmol/µg protein) in C3H10T1/2 cells

Source of variation	Sum of square	Degree of freedom	Mean square	F statistic	*p*-value	F critical value
Between groups	9.5604	4	2.3901	186.3058	1.0081×10^{-20}	2.6896
Within groups	0.3849	30	0.0128			
Total	9.9453	34				

Table S18: Descriptive statistics and Cohen's *d* of absorbance at 550 nm in Alizarin red S staining in C3H10T1/2 cells

Sample	C3H10T1/2	Bio2.5	Bio5	Bio10	Bio20
Average	0.1112	0.2343	0.4422	0.7797	1.4360
Standard deviation	0.0139	0.0396	0.0797	0.1221	0.2145
Margin of error for a 95% confidence interval	0.0146	0.0416	0.0837	0.1282	0.2251
Lower bound of 95% confidence interval	0.0966	0.1928	0.3585	0.6515	1.2109
Upper bound of 95% confidence interval	0.1258	0.2759	0.5259	0.9078	1.6611
Cohen's *d* (compared with C3H10T1/2)	Not applicable	4.1496	5.7827	7.6922	8.7174

Table S19: Shapiro–Wilk test of absorbance at 550 nm in Alizarin red S staining in C3H10T1/2 cells

Sample	C3H10T1/2	Bio2.5	Bio5	Bio10	Bio20
p-value	0.9888	0.2772	0.8833	0.5069	0.463
W	0.9732	0.8739	0.9518	0.9092	0.9036
B	0.03067	0.08279	0.174	0.2604	0.4559
Skewness	0.1771	0.8065	−0.4087	−0.1029	1.2021
Skewness shape	Potentially symmetrical (*p*-value = 0.834)	Potentially symmetrical (*p*-value = 0.34)	Potentially symmetrical (*p*-value = 0.629)	Potentially symmetrical (*p*-value = 0.903)	Potentially symmetrical (*p*-value = 0.155)
Excess kurtosis	−0.232	−0.5194	−1.2863	−1.5735	1.3239
Kurtosis Shape	Potentially Mesokurtic, normal like tails (*p*-value = 0.894)	Potentially Mesokurtic, normal like tails (*p*-value = 0.765)	Potentially Mesokurtic, normal like tails (*p*-value = 0.46)	Potentially Mesokurtic, normal like tails (*p*-value = 0.366)	Potentially Mesokurtic, normal like tails (*p*-value = 0.447)
Outliers	No outliers	No outliers	No outliers	No outliers	No outliers

Table S20: One-way analysis of variance of absorbance at 550 nm in Alizarin red S staining in C3H10T1/2 cells

Source of variation	Sum of square	Degree of freedom	Mean square	F statistic	*p*-value	F critical value
Between groups	6.7725	4	1.6931	122.6310	4.8031×10^{-16}	2.7587
Within groups	0.3452	25	0.0138			
Total	7.1177	29				

Table S21: Descriptive statistics and Cohen's *d* of absorbance at 570 nm in MTT assay in osteoclasts (OC)

Sample	OC	Bio1	Bio2.5	Bio5	Bio10	Bio20	Bio30
Average	0.4334	0.4286	0.4326	0.4105	0.4076	0.3943	0.1252
Standard deviation	0.0190	0.0236	0.0443	0.0225	0.0144	0.0565	0.0202
Margin of error for a 95% confidence interval	0.0159	0.0198	0.0370	0.0188	0.0120	0.0472	0.0169
Lower bound of 95% confidence interval	0.4174	0.4088	0.3955	0.3917	0.3955	0.3471	0.1084
Upper bound of 95% confidence interval	0.4493	0.4484	0.4696	0.4293	0.4196	0.4415	0.1421
Cohen's *d* (compared with OC)	Not applicable	−0.2219	−0.0235	−1.0946	−1.5301	−0.9271	−15.7089

Table S22: Shapiro–Wilk test of absorbance at 570 nm in MTT assay in osteoclasts (OC)

Sample	OC	Bio1	Bio2.5	Bio5	Bio10	Bio20	Bio30
p-value	0.4234	0.02953	0.3717	0.7175	0.4383	0.008838	0.07335
W	0.9144	0.7906	0.9081	0.9429	0.9161	0.7295	0.8337
B	0.04816	0.05563	0.1116	0.05781	0.03637	0.1276	0.04874
Skewness	−0.1091	−0.5627	−1.0087	−0.1267	0.3157	0.002795	−0.3946
Skewness shape	Potentially symmetrical (*p*-value = 0.885)	Potentially symmetrical (*p*-value = 0.454)	Potentially symmetrical (*p*-value = 0.18)	Potentially symmetrical (*p*-value = 0.866)	Potentially symmetrical (*p*-value = 0.675)	Potentially symmetrical (*p*-value = 0.997)	Potentially symmetrical (*p*-value = 0.6)
Excess kurtosis	2.4598	−2.089	0.9723	−0.8431	−1.5992	−2.7511	−2.0808
Kurtosis shape	Potentially Mesokurtic, normal like tails (*p*-value = 0.097)	Potentially Mesokurtic, normal like tails (*p*-value = 0.158)	Potentially Mesokurtic, normal like tails (*p*-value = 0.511)	Potentially Mesokurtic, normal like tails (*p*-value = 0.569)	Potentially Mesokurtic, normal like tails (*p*-value = 0.28)	Potentially Mesokurtic, normal like tails (*p*-value = 0.063)	Potentially Mesokurtic, normal like tails (*p*-value = 0.16)
Outliers	0.3983, 0.4671	No outliers	No outliers	No outliers	No outliers	No outliers	No outliers

Table S23: Descriptive statistics and Cohen's *d* of TRAP activities in osteoclasts (OC)

Sample	Bio2.5	Bio5	Bio10	Bio20
Average	80.5333	53.0000	31.4333	15.3333
Standard deviation	4.0796	3.9154	4.7931	5.3519
Margin of error for a 95% confidence interval	10.1343	9.7263	11.9066	13.2950
Lower bound of 95% confidence interval	70.3990	43.2737	19.5267	2.0384
Upper bound of 95% confidence interval	90.6677	62.7263	43.3399	28.6283
Cohen's *d* (compared with OC)	−6.7482	−16.9763	−20.2309	−22.3726

Table S24: Shapiro–Wilk test of TRAP activities in osteoclasts (OC)

Sample	Bio2.5	Bio5	Bio10	Bio20
p-value	1	0.796	0.1603	0.2446
W	0.9855	0.9173	0.7678	0.8044
B	5.7275	5.3032	5.9396	6.7882
Skewness	−0.613	−1.3294	−1.7287	1.6988
Skewness shape	Potentially symmetrical (*p*-value = 0.617)	Potentially symmetrical (*p*-value = 0.278)	Potentially symmetrical (*p*-value = 0.158)	Potentially symmetrical (*p*-value = 0.165)
Outliers	No outliers	No outliers	No outliers	No outliers

Table S25: One-way analysis of variance of TRAP activities in osteoclasts (OC)

Source of variation	Sum of square	Degree of freedom	Mean square	F statistic	*p*-value	F critical value
Between groups	1.4412	4	0.3603	215.5236	1.7805×10^{-9}	3.4780
Within groups	0.0167	10	0.0017			
Total	1.4580	14				

Table S26: Descriptive statistics and Cohen's *d* of percentage of pit area in osteoclasts (OC)

Sample	OC	OC+PC	OC+Bio20
Average	38.2973	32.9320	34.1613
Standard deviation	3.8338	2.6570	1.9693
Margin of error for a 95% confidence interval	9.5236	6.6004	4.8921
Lower bound of 95% confidence interval	28.7737	26.3316	29.2692
Upper bound of 95% confidence interval	47.8209	39.5324	39.0534
Cohen's *d* (compared with OC)	Not applicable	−1.6267	−1.3571

Table S27: Shapiro–Wilk test of percentage of pit area in osteoclasts (OC)

Sample	OC	OC+PC	OC+Bio20
p-value	0.8919	0.3576	0.2799
W	0.9344	0.8374	0.816
B	5.241	3.4386	2.5159
Skewness	1.214	1.6409	1.682
Skewness shape	Potentially symmetrical (*p*-value = 0.322)	Potentially symmetrical (*p*-value = 0.18)	Potentially symmetrical (*p*-value = 0.17)
Outliers	No outliers	No outliers	No outliers

Table S28: One-way analysis of variance of percentage of pit area in osteoclasts (OC)

Source of variation	Sum of square	Degree of freedom	Mean square	F statistic	*p*-value	F critical value
Between groups	47.4046	2	23.7023	2.7737	0.1403	5.1433
Within groups	51.2718	6	8.5453			
Total	98.6763	8				

Table S29: Descriptive statistics and Cohen's *d* of ROS production in osteoclasts (OC)

Sample	Bio2.5	Bio5	Bio10	Bio20
Average	89.0000	69.6667	49.3333	26.3333
Standard deviation	4.0497	8.9592	9.5638	6.8020
Margin of error for a 95% confidence interval	4.2499	9.4021	10.0366	7.1382
Lower bound of 95% confidence interval	84.7501	60.2646	39.2967	19.1951
Upper bound of 95% confidence interval	93.2499	79.0687	59.3699	33.4716
Cohen's *d* (compared with OC)	−3.8414	−4.7881	−7.4921	−15.3162

Table S30: Shapiro–Wilk test of ROS production in osteoclasts (OC)

Sample	Bio2.5	Bio5	Bio10	Bio20
p-value	0.2953	0.4156	0.6934	0.5644
W	0.8775	0.8972	0.9302	0.916
B	8.4827	18.9753	20.6253	14.5568
Skewness	−0.8131	0.03152	−1.0493	0.8393
Skewness shape	Potentially symmetrical (*p*-value = 0.336)	Potentially symmetrical (*p*-value = 0.97)	Potentially symmetrical (*p*-value = 0.214)	Potentially symmetrical (*p*-value = 0.321)
Excess kurtosis	−1.2894	−2.1879	0.9577	−0.5771
Kurtosis shape	Potentially Mesokurtic, normal like tails (*p*-value = 0.459)	Potentially Mesokurtic, normal like tails (*p*-value = 0.209)	Potentially M esokurtic, normal like tails (*p*-value = 0.582)	Potentially Mesokurtic, normal like tails (*p*-value = 0.74)
Outliers	No outliers	No outliers	No outliers	No outliers

Table S31: One-way analysis of variance of ROS production in osteoclasts (OC)

Source of variation	Sum of square	Degree of freedom	Mean square	F statistic	*p*-value	F critical value
Between groups	21275.4667	4	5318.8667	113.4571	1.2045×10^{-15}	2.7587
Within groups	1172.0000	25	46.8800			
Total	22447.4667	29				

Table S32: Descriptive statistics and Cohen's *d* of the relative expression of NF-κB p65 (normalized with β-actin) in osteoclasts

Sample	Bio1	Bio2.5	Bio5	Bio10	Bio20
Average	1.0000	0.4711	0.3034	0.0825	0.0243
Standard deviation	0.0000	0.2926	0.1771	0.0444	0.0392
Margin of error for a 95% confidence interval	0.7269	0.4399	0.1103	0.0368	0.0975
Lower bound of 95% confidence interval	−0.2557	−0.1366	−0.0278	0.0706	−0.0732
Upper bound of 95% confidence interval	1.1980	0.7433	0.1928	0.1442	0.1218
Cohen's *d* (compared with OC)	−2.5560	−5.5627	−29.2139	−85.2082	−35.1588

Table S33: Shapiro–Wilk test of the relative expression of NF-κB p65 (normalized with β-actin) in osteoclasts

Sample	Bio1	Bio2.5	Bio5	Bio10	Bio20
p-value	0.9995	0.2557	0.9733	0.6597	0.1569
W	0.9795	0.8082	0.9558	0.8953	0.766
B	0.4096	0.2252	0.06141	0.01982	0.04858
Skewness	−0.723	1.6937	1.0282	−1.4464	1.7293
Skewness shape	Potentially symmetrical (*p*-value = 0.555)	Potentially symmetrical (*p*-value = 0.167)	Potentially symmetrical (*p*-value = 0.401)	Potentially symmetrical (*p*-value = 0.238)	Potentially symmetrical (*p*-value = 0.158)
Outliers	No outliers	No outliers	No outliers	No outliers	No outliers

Table S34: One-way analysis of variance of the relative expression of NF-κB p65 (normalized with β-actin) in osteoclasts

Source of variation	Sum of square	Degree of freedom	Mean square	F statistic	*p*-value	F critical value
Between groups	2.0213	5	0.4043	20.0923	1.8743×10^{-5}	3.1059
Within groups	0.2414	12	0.0201			
Total	2.2627	17				

Table S35: Descriptive statistics and Cohen's *d* of the expression of pri-miR-21 in osteoclasts (OC)

Sample	Bio2.5	Bio5	Bio10	Bio20
Average	0.8360	0.7337	0.5123	0.2567
Standard deviation	0.1357	0.1068	0.0995	0.0440
Margin of error for a 95% confidence interval	0.3372	0.2653	0.2472	0.1093
Lower bound of 95% confidence interval	0.4988	0.4683	0.2652	0.1474
Upper bound of 95% confidence interval	1.1732	0.9990	0.7595	0.3660
Cohen's *d* (compared with OC)	−1.7088	−3.5264	−6.9313	−23.8896

Table S36: Shapiro–Wilk test of the expression of pri-miR-21 in osteoclasts (OC)

Sample	Bio2.5	Bio5	Bio10	Bio20
p-value	0.9998	0.5479	1	1
W	0.982	0.8765	1	0.9998
B	0.1902	0.1414	0.1407	0.06222
Skewness	0.6796	−1.5252	0.01508	0.06816
Skewness shape	Potentially symmetrical (*p*-value = 0.579)	Potentially symmetrical (*p*-value = 0.213)	Potentially symmetrical (*p*-value = 0.99)	Potentially symmetrical (*p*-value = 0.956)
Outliers	No outliers	No outliers	No outliers	No outliers

Table S37: One-way analysis of variance of the expression of pri-miR-21 in osteoclasts (OC)

Source of variation	Sum of square	Degree of freedom	Mean square	F statistic	*p*-value	F critical value
Between groups	1.0086	4	0.2521	30.2573	1.4547×10^{-5}	3.4780
Within groups	0.0833	10	0.0083			
Total	1.0919	14				

Table S38: Descriptive statistics and Cohen's *d* of the expression of pre-miR-21 in osteoclasts (OC)

Sample	Bio2.5	Bio5	Bio10	Bio20
Average	0.9417	0.8297	0.4527	0.2673
Standard deviation	0.0514	0.1044	0.1223	0.0958
Margin of error for a 95% confidence interval	0.1277	0.2592	0.3039	0.2381
Lower bound of 95% confidence interval	0.8140	0.5704	0.1488	0.0292
Upper bound of 95% confidence interval	1.0693	1.0889	0.7565	0.5054
Cohen's *d* (compared with OC)	−1.6052	−2.3083	−6.3282	−10.8106

Table S39: Shapiro–Wilk test of the expression of pre-miR-21 in osteoclasts (OC)

Sample	Bio2.5	Bio5	Bio10	Bio20
p-value	0.1581	0.7454	0.3051	0.1447
W	0.7666	0.9091	0.8235	0.759
B	0.06364	0.1407	0.157	0.1181
Skewness	1.7291	−1.3768	−1.6692	1.7312
Skewness shape	Potentially symmetrical (*p*-value = 0.158)	Potentially symmetrical (*p*-value = 0.261)	Potentially symmetrical (*p*-value = 0.173)	Potentially symmetrical (*p*-value = 0.158)
Outliers	No outliers	No outliers	No outliers	No outliers

Table S40: One-way analysis of variance of the expression of pre-miR-21 in osteoclasts (OC)

Source of variation	Sum of square	Degree of freedom	Mean square	F statistic	*p*-value	F critical value
Between groups	1.2407	4	0.3102	41.1607	3.5169×10^{-6}	3.4780
Within groups	0.0754	10	0.0075			
Total	1.3161	14				

Table S41: Descriptive statistics and Cohen's *d* of the expression of mature-miR-21 in osteoclasts (OC)

Sample	Bio2.5	Bio5	Bio10	Bio20
Average	0.7483	0.5287	0.2540	0.1743
Standard deviation	0.0629	0.1046	0.0510	0.0794
Margin of error for a 95% confidence interval	0.1563	0.2598	0.1268	0.1972
Lower bound of 95% confidence interval	0.5920	0.2689	0.1272	−0.0229
Upper bound of 95% confidence interval	0.9047	0.7885	0.3808	0.3715
Cohen's *d* (compared with OC)	−5.6555	−6.3739	−20.6744	−14.7085

Table S42: Shapiro–Wilk test of the expression of mature-miR-21 in osteoclasts (OC)

Sample	Bio2.5	Bio5	Bio10	Bio20
p-value	0.1305	0.7781	0.5957	0.7165
W	0.75	0.9144	0.8848	0.9044
B	0.07707	0.1414	0.06788	0.1068
Skewness	1.7321	−1.3468	1.4928	1.4015
Skewness shape	Potentially symmetrical (*p*-value = 0.157)	Potentially symmetrical (*p*-value = 0.271)	Potentially symmetrical (*p*-value = 0.223)	Potentially symmetrical (*p*-value = 0.252)
Outliers	No outliers	No outliers	No outliers	No outliers

Table S43: One-way analysis of variance of the expression of mature-miR-21 in osteoclasts (OC)

Source of variation	Sum of square	Degree of freedom	Mean square	F statistic	*p*-value	F critical value
Between groups	1.4119	4	0.3530	74.1451	2.1551×10^{-7}	3.4780
Within groups	0.0476	10	0.0048			
Total	1.4595	14				

Table S44: Descriptive statistics and Cohen's *d* of TRAP activity in osteoclasts (OC) transfected with an miR-21 mimic

Sample	Bio2.5	Bio5	Bio10	Bio20
Average	1.2590	1.9597	3.8487	6.1530
Standard deviation	0.0501	0.1774	0.5541	0.9554
Margin of error for a 95% confidence interval	0.1245	0.4406	1.3765	2.3732
Lower bound of 95% confidence interval	1.1345	1.5191	2.4722	3.7798
Upper bound of 95% confidence interval	1.3835	2.4003	5.2251	8.5262
Cohen's *d* (compared with OC)	7.3081	7.6516	7.2705	7.6280

Table S45: Shapiro–Wilk test of TRAP activity in osteoclasts (OC) transfected with an miR-21 mimic

Sample	Bio2.5	Bio5	Bio10	Bio20
p-value	1	0.9422	0.2847	1
W	0.9952	0.9458	0.8175	0.9992
B	0.07071	0.2439	0.7085	1.3506
Skewness	0.3569	1.122	−1.6796	0.1412
Skewness shape	Potentially symmetrical (*p*-value = 0.771)	Potentially symmetrical (*p*-value = 0.36)	Potentially symmetrical (*p*-value = 0.17)	Potentially symmetrical (*p*-value = 0.908)
Outliers	No outliers	No outliers	No outliers	No outliers

Table S46: One-way analysis of variance of TRAP activity in osteoclasts (OC) transfected with an miR-21 mimic

Source of variation	Sum of square	Degree of freedom	Mean square	F statistic	*p*-value	F critical value
Between groups	55.9603	4	13.9901	55.7949	8.3926×10^{-7}	3.4780
Within groups	2.5074	10	0.2507			
Total	58.4677	14				

Table S47: Descriptive statistics and Cohen's *d* of CTSK activity in osteoclasts (OC) transfected with an miR-21 mimic

Sample	Bio2.5	Bio5	Bio10	Bio20
Average	1.1663	2.4197	4.8137	7.2623
Standard deviation	0.0583	0.6405	0.8230	1.4366
Margin of error for a 95% confidence interval	0.1449	1.5912	2.0445	3.5688
Lower bound of 95% confidence interval	1.0215	0.8285	2.7692	3.6935
Upper bound of 95% confidence interval	1.3112	4.0109	6.8582	10.8311
Cohen's *d* (compared with OC)	4.0334	3.1344	6.5531	6.1646

Table S48: Shapiro–Wilk test of CTSK activity in osteoclasts (OC) transfected with an miR-21 mimic

Sample	Bio2.5	Bio5	Bio10	Bio20
p-value	0.1545	0.3174	1	1
W	0.7647	0.827	0.9975	0.9908
B	0.07212	0.8238	1.1625	2.0223
Skewness	−1.7298	1.6628	−0.2579	0.4929
Skewness shape	Potentially symmetrical (*p*-value = 0.158)	Potentially symmetrical (*p*-value = 0.175)	Potentially symmetrical (*p*-value = 0.833)	Potentially symmetrical (*p*-value = 0.687)
Outliers	No outliers	No outliers	No outliers	No outliers

Table S49: One-way analysis of variance of CTSK activity in osteoclasts (OC) transfected with an miR-21 mimic

Source of variation	Sum of square	Degree of freedom	Mean square	F statistic	*p*-value	F critical value
Between groups	85.8106	4	21.4527	33.9978	8.5308×10^{-6}	3.4780
Within groups	6.3100	10	0.6310			
Total	92.1206	14				

Table S50: Descriptive statistics and Cohen's *d* of TRAP activity in osteoclasts (OC) transfected with an miR-21 inhibitor

Sample	Bio2.5	Bio5	Bio10	Bio20
Average	0.8887	0.7650	0.4920	0.2730
Standard deviation	0.0690	0.0461	0.0711	0.0543
Margin of error for a 95% confidence interval	0.1715	0.1146	0.1767	0.1349
Lower bound of 95% confidence interval	0.7172	0.6504	0.3153	0.1381
Upper bound of 95% confidence interval	1.0601	0.8796	0.6687	0.4079
Cohen's *d* (compared with OC)	−2.2811	−7.2044	−10.0986	−18.9391

Table S51: Shapiro–Wilk test of TRAP activity in osteoclasts (OC) transfected with an miR-21 inhibitor

Sample	Bio2.5	Bio5	Bio10	Bio20
p-value	0.7776	0.7496	0.9213	0.4871
W	0.9143	0.9098	0.9407	0.8654
B	0.09334	0.06222	0.09758	0.07142
Skewness	−1.3474	−1.373	−1.1651	−1.5643
Skewness shape	Potentially symmetrical (*p*-value = 0.271)	Potentially symmetrical (*p*-value = 0.262)	Potentially symmetrical (*p*-value = 0.341)	Potentially symmetrical (*p*-value = 0.202)
Outliers	No outliers	No outliers	No outliers	No outliers

Table S52: One-way analysis of variance of TRAP activity in osteoclasts (OC) transfected with an miR-21 inhibitor

Source of variation	Sum of square	Degree of freedom	Mean square	F statistic	*p*-value	F critical value
Between groups	1.0623	4	0.2656	89.1146	8.8723×10^{-8}	3.4780
Within groups	0.0298	10	0.0030			
Total	1.0921	14				

Table S53: Descriptive statistics and Cohen's *d* of CTSK activity in osteoclasts (OC) transfected with an miR-21 inhibitor

Sample	Bio2.5	Bio5	Bio10	Bio20
Average	0.9813	0.8080	0.4127	0.2410
Standard deviation	0.0523	0.0945	0.1025	0.1460
Margin of error for a 95% confidence interval	0.1298	0.2348	0.2546	0.3628
Lower bound of 95% confidence interval	0.8515	0.5732	0.1580	−0.1218
Upper bound of 95% confidence interval	1.1111	1.0428	0.6673	0.6038
Cohen's *d* (compared with OC)	−0.5052	−2.8722	−8.1028	−7.3506

Table S54: Shapiro–Wilk test of CTSK activity in osteoclasts (OC) transfected with an miR-21 inhibitor

Sample	Bio2.5	Bio5	Bio10	Bio20
p-value	0.1435	1	1	0.9971
W	0.7582	0.9992	0.9998	0.9724
B	0.06435	0.1336	0.145	0.2036
Skewness	−1.7313	−0.1427	0.07314	0.8311
Skewness shape	Potentially symmetrical (*p*-value = 0.157)	Potentially symmetrical (*p*-value = 0.907)	Potentially symmetrical (*p*-value = 0.952)	Potentially symmetrical (*p*-value = 0.497)
Outliers	No outliers	No outliers	No outliers	No outliers

Table S55: One-way analysis of variance of CTSK activity in osteoclasts (OC) transfected with an miR-21 inhibitor

Source of variation	Sum of square	Degree of freedom	Mean square	F statistic	*p*-value	F critical value
Between groups	1.4202	4	0.3551	40.8110	3.6597×10^{-6}	3.4780
Within groups	0.0870	10	0.0087			
Total	1.5072	14				

Table S56: Descriptive statistics and Cohen's *d* of the relative expression of PTEN in osteoclasts (standard RANKL-supplemented differentiation medium)

Sample	Bio2.5	Bio5	Bio10	Bio20
Average	1.0450	1.2240	2.5300	3.5897
Standard deviation	0.0679	0.1149	0.4486	0.4714
Margin of error for a 95% confidence interval	0.1687	0.2854	1.1144	1.1711
Lower bound of 95% confidence interval	0.8763	0.9386	1.4156	2.4186
Upper bound of 95% confidence interval	1.2137	1.5094	3.6444	4.7607
Cohen's *d* (compared with OC)	0.9371	2.7571	4.8233	7.7688

Table S57: Shapiro–Wilk test of the relative expression of PTEN in osteoclasts (standard RANKL-supplemented differentiation medium)

Sample	Bio2.5	Bio5	Bio10	Bio20
p-value	0.3419	1	0.9995	0.1405
W	0.8335	0.9931	0.9796	0.7564
B	0.08768	0.1619	0.6279	0.5798
Skewness	1.6496	0.4269	0.7221	1.7316
Skewness shape	Potentially symmetrical (*p*-value = 0.178)	Potentially symmetrical (*p*-value = 0.727)	Potentially symmetrical (*p*-value = 0.555)	Potentially symmetrical (*p*-value = 0.157)
Outliers	No outliers	No outliers	No outliers	No outliers

Table S58: One-way analysis of variance of the relative expression of PTEN in osteoclasts (standard RANKL-supplemented differentiation medium)

Source of variation	Sum of square	Degree of freedom	Mean square	F statistic	*p*-value	F critical value
Between groups	15.7422	4	3.9355	44.5912	2.4183×10^{-6}	3.4780
Within groups	0.8826	10	0.0883			
Total	16.6248	14				

Table S59: Descriptive statistics and Cohen's *d* of the expression of pri-miR-21 in PTEN-KD osteoclasts (OC)

Sample	Bio2.5	Bio5	Bio10	Bio20
Average	0.8930	0.7817	0.5793	0.2397
Standard deviation	0.0737	0.0525	0.0574	0.1108
Margin of error for a 95% confidence interval	0.1830	0.1305	0.1427	0.2753
Lower bound of 95% confidence interval	0.7100	0.6511	0.4366	−0.0356
Upper bound of 95% confidence interval	1.0760	0.9122	0.7220	0.5150
Cohen's *d* (compared with OC)	−2.0541	−5.8759	−10.3556	−9.7032

Table S60: Shapiro–Wilk test of the expression of pri-miR-21 in PTEN-KD osteoclasts (OC)

Sample	Bio2.5	Bio5	Bio10	Bio20
p-value	0.9719	0.1574	0.1422	0.2498
W	0.9552	0.7663	0.7575	0.8062
B	0.1018	0.06505	0.07071	0.1407
Skewness	−1.0339	−1.7292	−1.7315	−1.6964
Skewness shape	Potentially symmetrical (*p*-value = 0.399)	Potentially symmetrical (*p*-value = 0.158)	Potentially symmetrical (*p*-value = 0.157)	Potentially symmetrical (*p*-value = 0.166)
Outliers	No outliers	No outliers	No outliers	No outliers

Table S61: One-way analysis of variance of the expression of pri-miR-21 in PTEN-KD osteoclasts (OC)

Source of variation	Sum of square	Degree of freedom	Mean square	F statistic	*p*-value	F critical value
Between groups	1.0811	4	0.2703	56.8562	7.6736×10^{-7}	3.4780
Within groups	0.0475	10	0.0048			
Total	1.1287	14				

Table S62: Descriptive statistics and Cohen's *d* of the expression of pre-miR-21 in PTEN-KD osteoclasts (OC)

Sample	Bio2.5	Bio5	Bio10	Bio20
Average	0.8380	0.7140	0.5153	0.2863
Standard deviation	0.0649	0.0630	0.1040	0.0657
Margin of error for a 95% confidence interval	0.1611	0.1564	0.2584	0.1633
Lower bound of 95% confidence interval	0.6769	0.5576	0.2569	0.1231
Upper bound of 95% confidence interval	0.9991	0.8704	0.7738	0.4496
Cohen's *d* (compared with OC)	−3.5322	−6.4241	−6.5881	−15.3551

Table S63: Shapiro–Wilk test of the expression of pre-miR-21 in PTEN-KD osteoclasts (OC)

Sample	Bio2.5	Bio5	Bio10	Bio20
p-value	0.5119	0.9123	1	0.3992
W	0.87	0.9387	0.9992	0.8472
B	0.08556	0.08627	0.1471	0.08556
Skewness	1.5486	−1.1814	0.144	−1.6173
Skewness shape	Potentially symmetrical (*p*-value = 0.206)	Potentially symmetrical (*p*-value = 0.335)	Potentially symmetrical (*p*-value = 0.906)	Potentially symmetrical (*p*-value = 0.187)
Outliers	No outliers	No outliers	No outliers	No outliers

Table S64: One-way analysis of variance of the expression of pre-miR-21 in PTEN-KD osteoclasts (OC)

Source of variation	Sum of square	Degree of freedom	Mean square	F statistic	*p*-value	F critical value
Between groups	0.9305	4	0.2326	49.8880	1.4258×10^{-6}	3.4780
Within groups	0.0466	10	0.0047			
Total	0.9772	14				

Table S65: Descriptive statistics and Cohen's *d* of the expression of mature-miR-21 in PTEN-KD osteoclasts (OC)

Sample	Bio2.5	Bio5	Bio10	Bio20
Average	0.8327	0.6407	0.3787	0.1383
Standard deviation	0.1275	0.1061	0.0569	0.0648
Margin of error for a 95% confidence interval	0.3167	0.2636	0.1413	0.1610
Lower bound of 95% confidence interval	0.5160	0.3771	0.2374	−0.0227
Upper bound of 95% confidence interval	1.1493	0.9042	0.5199	0.2994
Cohen's *d* (compared with OC)	−1.8564	−4.7896	−15.4507	−18.7979

Table S66: Shapiro–Wilk test of the expression of mature-miR-21 in PTEN-KD osteoclasts (OC)

Sample	Bio2.5	Bio5	Bio10	Bio20
p-value	1	1	0.1424	0.5997
W	0.9926	0.9981	0.7576	0.8854
B	0.1796	0.1499	0.07	0.08627
Skewness	0.4427	−0.2256	−1.7314	1.49
Skewness shape	Potentially symmetrical (*p*-value = 0.718)	Potentially symmetrical (*p*-value = 0.854)	Potentially symmetrical (*p*-value = 0.157)	Potentially symmetrical (*p*-value = 0.224)
Outliers	No outliers	No outliers	No outliers	No outliers

Table S67: One-way analysis of variance of the expression of mature-miR-21 in PTEN-KD osteoclasts (OC)

Source of variation	Sum of square	Degree of freedom	Mean square	F statistic	*p*-value	F critical value
Between groups	1.4337	4	0.3584	51.2859	1.2513×10^{-6}	3.4780
Within groups	0.0699	10	0.0070			
Total	1.5036	14				

Table S68: Descriptive statistics and Cohen's *d* of the relative NFATc1 expression in PTEN-KD osteoclasts (OC) transfected with an miR-21 mimic

Sample	Bio2.5	Bio5	Bio10	Bio20
Average	1.1153	1.2863	3.1263	6.1230
Standard deviation	0.0997	0.0445	0.9950	1.0010
Margin of error for a 95% confidence interval	0.2477	0.1104	2.4717	2.4866
Lower bound of 95% confidence interval	0.8676	1.1759	0.6546	3.6364
Upper bound of 95% confidence interval	1.3631	1.3968	5.5981	8.6096
Cohen's *d* (compared with OC)	1.6356	9.1087	3.0222	7.2378

Table S69: Shapiro–Wilk test of the relative NFATc1 expression in PTEN-KD osteoclasts (OC) transfected with an miR-21 mimic

Sample	Bio2.5	Bio5	Bio10	Bio20
p-value	1	0.1305	1	1
W	0.9955	0.75	1	1
B	0.1407	0.05445	1.4071	1.4156
Skewness	−0.3439	−1.7321	0.01508	-3.99×10^{-15}
Skewness shape	Potentially symmetrical (*p*-value = 0.718)	Potentially symmetrical (*p*-value = 0.854)	Potentially symmetrical (*p*-value = 0.157)	Potentially symmetrical (*p*-value = 0.224)
Outliers	No outliers	No outliers	No outliers	No outliers

Table S70: One-way analysis of variance of the relative NFATc1 expression in PTEN-KD osteoclasts (OC) transfected with an miR-21 mimic

Source of variation	Sum of square	Degree of freedom	Mean square	F statistic	*p*-value	F critical value
Between groups	57.4625	4	14.3656	35.8432	6.6857×10^{-6}	3.4780
Within groups	4.0079	10	0.4008			
Total	61.4704	14				

Table S71: Descriptive statistics and Cohen's *d* of the relative NFATc1 expression in PTEN-KD osteoclasts (OC) transfected with an miR-21 inhibitor

Sample	Bio2.5	Bio5	Bio10	Bio20
Average	0.9017	0.8043	0.4350	0.2543
Standard deviation	0.0854	0.0950	0.1084	0.0690
Margin of error for a 95% confidence interval	0.2121	0.2361	0.2694	0.1714
Lower bound of 95% confidence interval	0.6896	0.5682	0.1656	0.0829
Upper bound of 95% confidence interval	1.1138	1.0404	0.7044	0.4258
Cohen's *d* (compared with OC)	−1.6287	−2.9114	−7.3691	−15.2809

Table S72: Shapiro–Wilk test of the relative NFATc1 expression in PTEN-KD osteoclasts (OC) transfected with an miR-21 inhibitor

Sample	Bio2.5	Bio5	Bio10	Bio20
p-value	1	1	0.455	0.1616
W	0.991	0.9991	0.8591	0.7686
B	0.1202	0.1343	0.1421	0.08556
Skewness	−0.486	0.1576	−1.5841	1.7284
Skewness shape	Potentially symmetrical (*p*-value = 0.691)	Potentially symmetrical (*p*-value = 0.898)	Potentially symmetrical (*p*-value = 0.196)	Potentially symmetrical (*p*-value = 0.158)
Outliers	No outliers	No outliers	No outliers	No outliers

Table S73: One-way analysis of variance of the relative NFATc1 expression in PTEN-KD osteoclasts (OC) transfected with an miR-21 inhibitor

Source of variation	Sum of square	Degree of freedom	Mean square	F statistic	*p*-value	F critical value
Between groups	1.2246	4	0.3062	46.6090	1.9644×10^{-6}	3.4780
Within groups	0.0657	10	0.0066			
Total	1.2903	14				

Table S74: Descriptive statistics and Cohen's *d* of the relative expression of PTEN in osteoclasts (standard medium enriched with 10% (*v*/*v*) OCM)

Sample	Bio2.5	Bio5	Bio10	Bio20
Average	1.3220	2.2867	4.0100	6.3563
Standard deviation	0.1646	0.3900	0.3017	0.7673
Margin of error for a 95% confidence interval	0.4089	0.9687	0.7495	1.9062
Lower bound of 95% confidence interval	0.9131	1.3180	3.2605	4.4501
Upper bound of 95% confidence interval	1.7309	3.2554	4.7595	8.2625
Cohen's *d* (compared with OC)	2.7662	4.6663	14.1088	9.8716

Table S75: Shapiro–Wilk test of the relative expression of PTEN in osteoclasts (standard medium enriched with 10% (*v*/*v*) OCM)

Sample	Bio2.5	Bio5	Bio10	Bio20
p-value	0.1758	0.4335	0.8703	0.8755
W	0.7758	0.8546	0.9302	0.9312
B	0.2051	0.5098	0.4115	1.0472
Skewness	1.7249	1.5971	−1.2446	1.2376
Skewness shape	Potentially symmetrical (*p*-value = 0.159)	Potentially symmetrical (*p*-value = 0.192)	Potentially symmetrical (*p*-value = 0.31)	Potentially symmetrical (*p*-value = 0.312)
Outliers	No outliers	No outliers	No outliers	No outliers

Table S76: One-way analysis of variance of the relative expression of PTEN in osteoclasts (standard medium enriched with 10% (*v*/*v*) OCM)

Source of variation	Sum of square	Degree of freedom	Mean square	F statistic	*p*-value	F critical value
Between groups	58.8284	4	14.7071	85.6044	1.0776×10^{-7}	3.4780
Within groups	1.7180	10	0.1718			
Total	60.5465	14				

Table S77: Descriptive statistics and Cohen's *d* of the relative expression of PDCD4 in osteoclasts (standard RANKL-supplemented differentiation medium)

Sample	Bio2.5	Bio5	Bio10	Bio20
Average	1.0693	1.8120	2.9120	4.6917
Standard deviation	0.0747	0.1005	0.6082	0.6290
Margin of error for a 95% confidence interval	0.1856	0.2497	1.5108	1.5625
Lower bound of 95% confidence interval	0.8837	1.5623	1.4012	3.1291
Upper bound of 95% confidence interval	1.2549	2.0617	4.4228	6.2542
Cohen's *d* (compared with OC)	1.3123	11.4259	4.4459	8.3002

Table S78: Shapiro–Wilk test of the relative expression of PDCD4 in osteoclasts (standard RANKL-supplemented differentiation medium)

Sample	Bio2.5	Bio5	Bio10	Bio20
p-value	0.4866	1	0.2808	0.4197
W	0.8653	0.9999	0.8163	0.8517
B	0.09829	0.1421	0.7771	0.8209
Skewness	−1.5646	−0.04477	1.6816	1.6053
Skewness shape	Potentially symmetrical (*p*-value = 0.201)	Potentially symmetrical (*p*-value = 0.971)	Potentially symmetrical (*p*-value = 0.17)	Potentially symmetrical (*p*-value = 0.19)
Outliers	No outliers	No outliers	No outliers	No outliers

Table S79: One-way analysis of variance of the relative expression of PDCD4 in osteoclasts (standard RANKL-supplemented differentiation medium)

Source of variation	Sum of square	Degree of freedom	Mean square	F statistic	*p*-value	F critical value
Between groups	28.6118	4	7.1529	45.7803	2.1372×10^{-6}	3.4780
Within groups	1.5624	10	0.1562			
Total	30.1742	14				

Table S80: Descriptive statistics and Cohen's *d* of the relative expression of PDCD4 in osteoclasts (standard medium enriched with 10% (*v*/*v*) OCM)

Sample	Bio2.5	Bio5	Bio10	Bio20
Average	1.9007	4.0060	4.7847	6.7397
Standard deviation	0.0826	0.5293	0.6300	0.4602
Margin of error for a 95% confidence interval	0.2052	1.3148	1.5649	1.1432
Lower bound of 95% confidence interval	1.6955	2.6912	3.2197	5.5965
Upper bound of 95% confidence interval	2.1058	5.3208	6.3496	7.8828
Cohen's *d* (compared with OC)	15.4233	8.0318	8.4962	17.6386

Table S81: Shapiro–Wilk test of the relative expression of PDCD4 in osteoclasts (standard medium enriched with 10% (*v*/*v*) OCM)

Sample	Bio2.5	Bio5	Bio10	Bio20
p-value	1	0.1646	0.1526	0.9995
W	0.9859	0.7702	0.7636	0.9797
B	0.116	0.6569	0.7785	0.6442
Skewness	−0.6059	1.7277	1.7301	0.7199
Skewness shape	Potentially symmetrical (*p*-value = 0.621)	Potentially symmetrical (*p*-value = 0.158)	Potentially symmetrical (*p*-value = 0.158)	Potentially symmetrical (*p*-value = 0.557)
Outliers	No outliers	No outliers	No outliers	No outliers

Table S82: One-way analysis of variance of the relative expression of PDCD4 in osteoclasts (standard medium enriched with 10% (*v*/*v*) OCM)

Source of variation	Sum of square	Degree of freedom	Mean square	F statistic	*p*-value	F critical value
Between groups	63.1091	4	15.7773	88.0819	9.3872×10^{-8}	3.4780
Within groups	1.7912	10	0.1791			
Total	64.9003	14				
